# Multidimensional mechanisms of natural products in modulating metabolic dysfunction–associated kidney disease: recent advances and future perspectives

**DOI:** 10.3389/fphar.2026.1881128

**Published:** 2026-06-26

**Authors:** Yue Zhang, Jiarui Li, Qi Zhong, Mingda Han, Xiao Sun, Huili Huang, Shuang Zhao, Hao Xu, Kaile Ma, Xiangyan Li, Min Li

**Affiliations:** 1 Guang’anmen Hospital, China Academy of Chinese Medical Sciences, Beijing, China; 2 College of Traditional Chinese Medicine, Changchun University of Chinese Medicine, Changchun, China; 3 Research Laboratory of Molecular Biology, Guang’anmen Hospital, China Academy of Chinese Medical Sciences, Beijing, China

**Keywords:** diabetic kidney disease, hyperuricemic nephropathy, metabolic dysfunction–associated kidney disease, natural products, obesity-related glomerulopathy, targets

## Abstract

Metabolic dysfunction–associated kidney disease (MDAKD), encompassing diabetic kidney disease (DKD), obesity-related glomerulopathy (ORG), and hyperuricemic nephropathy (HN), has emerged as a major global health challenge alongside the increasing prevalence of metabolic syndrome. The development and progression of MDAKD are driven by a complex pathogenic network involving metabolic dysregulation, gut microbiota imbalance, oxidative stress, mitochondrial dysfunction, chronic inflammation, and renal fibrosis. Although previous reviews have focused on individual pathogenic mechanisms or specific disease subtypes, comprehensive syntheses integrating distinct MDAKD subtypes within a unified framework while systematically elucidating the multidimensional regulatory mechanisms of natural products remain limited. Natural products have attracted growing attention owing to their multi-component and multi-target pharmacological properties. Increasing evidence suggests that their therapeutic potential extends beyond the modulation of individual pathogenic pathways, encompassing the coordinated regulation of multiple interconnected pathological networks. Diverse bioactive constituents, including polysaccharides, flavonoids, phenolic acids, triterpenes, alkaloids, and saponins, have demonstrated considerable renoprotective effects. Compared with conventional single-pathway interventions, the multi-target regulatory characteristics of natural products, together with their generally favorable safety and tolerability profiles, make them particularly suited to the multifactorial nature of MDAKD. This review systematically summarizes the key pathogenic mechanisms underlying MDAKD and comprehensively discusses recent advances in natural product–based interventions from both mechanistic and pharmacological perspectives. Current evidence indicates that natural products exert renoprotective effects through the coordinated modulation of multiple interconnected pathological processes, thereby establishing a multidimensional regulatory pattern that closely aligns with the complex pathophysiology of MDAKD. Despite challenges related to limited bioavailability, incompletely characterized pharmacokinetic properties, insufficient safety evaluation, and the lack of high-quality clinical evidence, natural products remain promising candidates for the integrated management of MDAKD. Accordingly, this review proposes a multidimensional regulatory framework for natural product–mediated interventions in MDAKD, providing a theoretical basis for future mechanistic studies, drug development, and clinical research.

## 1 Introduction

### 1.1 MDAKD as a global health challenge

With the increasing global burden of metabolic disorders such as metabolic syndrome (MetS), metabolic dysfunction–associated kidney disease (MDAKD) has attracted widespread attention and has become an important public health issue. MDAKD is a term describing kidney diseases caused by metabolic dysfunction (Bansal and Chonchol, 2025), Including diabetic kidney disease (DKD), hyperuricemic nephropathy (HN), and obesity-related glomerulopathy (ORG), the prevalence of these conditions continues to rise with the global increase in MetS and aging populations. Recent epidemiological evidence indicates a steadily increasing global burden of MDAKD and its major subtypes. Analyses based on the Global Burden of Disease (GBD) 2021 database estimated that approximately 107.6 million individuals worldwide were living with DKD in 2021, accounting for nearly 477,300 deaths and underscoring its substantial contribution to chronic kidney disease and kidney failure (Zhang et al., 2025a). Concurrently, the growing prevalence of obesity and hyperuricemia has further increased the burden of ORG and HN (Martínez-Montoro et al., 2022). Notably, the global prevalence of hyperuricemia reached 11.2% in women and 18.6% in men by 2023 (Ngandeu-Singwe, 2003). Similar trends have also been observed in China, where the burden of DKD and hyperuricemia-associated kidney disease continues to rise (Hu et al., 2025; Im et al., 2025). Collectively, these epidemiological trends highlight MDAKD as an increasingly important public health challenge worldwide.

According to global statistics, approximately 10% of the population is affected by kidney diseases of varying severity, which constitute a major contributor to mortality and underscore the public health significance of renal disorders (Qu and Jiao, 2023). The pathogenesis of MDAKD is highly complex, as the kidney not only functions as a central organ for maintaining metabolic homeostasis but also serves as a primary target of metabolic dysregulation. MDAKD is driven by multifactorial mechanisms: chronic metabolic disturbances, insulin resistance, oxidative stress, and persistent inflammation can induce sustained damage to renal structure and function (Nakamura et al., 2023; Minami et al., 2024),characterized by glomerular filtration barrier disruption, tubular reabsorption impairment, and interstitial fibrosis, thereby contributing to MDAKD onset and progression.

### 1.2 Current therapeutic strategies for MDAKD: opportunities and challenges of natural product interventions

Despite recent advances in drug development for MDAKD and an expanded range of therapeutic options, the therapeutic efficacy of available agents remains limited due to the multifactorial and complex pathogenesis of the disease. Currently, the clinical management of MDAKD primarily relies on the control of metabolic risk factors and the use of renoprotective therapies. Renin–angiotensin–aldosterone system (RAAS) inhibitors, sodium–glucose cotransporter 2 (SGLT2) inhibitors, and glucagon-like peptide-1 receptor agonists (GLP-1RAs) have demonstrated substantial renal and metabolic benefits in the management of diabetes-associated chronic kidney disease, while urate-lowering agents are widely used for the treatment of hyperuricemia-related renal injury. Nevertheless, several limitations remain. SGLT2 inhibitors may increase the risk of genitourinary infections and ketoacidosis, whereas GLP-1RAs are often limited by gastrointestinal adverse effects and challenges in long-term treatment adherence (Qiu et al., 2021; Abasheva et al., 2024). Although uricase-based therapies exhibit potent urate-lowering efficacy, their clinical application is restricted by infusion-related reactions, immunogenicity, and limitations in specific patient populations (Schlesinger et al., 2022). In addition, disease-specific pharmacological options for obesity-related glomerulopathy remain limited. Therefore, although current therapies can improve metabolic, cardiovascular, and renal outcomes, they primarily target discrete pathogenic pathways and are insufficient to comprehensively modulate the complex and interconnected pathological networks underlying MDAKD, including oxidative stress, chronic inflammation, gut microbiota dysbiosis, and renal fibrosis.

Natural products are chemical constituents derived from plants, animals, microorganisms, and other biological sources (Jiang et al., 2025), garnering considerable attention due to their structural diversity, low toxicity, multi-target effects, and demonstrated bioactivity (Zeng et al., 2017). With ongoing advances in isolation, purification, and structural characterization techniques, an increasing number of pharmacologically active natural products have been identified and have become integral to drug discovery (Newman and Cragg, 2020). Accumulating evidence suggests that natural products represent a promising therapeutic avenue for MDAKD. In contrast to current therapies that primarily focus on discrete pathogenic pathways, their multi-component and multi-target characteristics may enable multidimensional modulation of the interconnected pathological networks underlying MDAKD, offering a therapeutic paradigm that is better aligned with its multifactorial disease nature. Nonetheless, several challenges remain, including complex chemical composition, low bioavailability, potential toxicity, absence of standardized dosing protocols, and limited clinical translational evidence. Therefore, there is an urgent need to develop safe and effective therapeutic strategies that can identify novel targets and implement sustainable interventions to mitigate MDAKD progression.

In this review, we systematically summarize the key pathogenic mechanisms underlying MDAKD and comprehensively synthesize current evidence on natural product–based interventions. Unlike previous reviews focusing on individual disease subtypes or isolated pathogenic pathways, this review integrates the shared pathological basis of DKD, ORG, and HN within the MDAKD framework and highlights the multidimensional regulatory mechanisms of natural products across interconnected disease networks. We further discuss current challenges and future research priorities, providing a conceptual framework for the rational development and application of natural products in MDAKD.

## 2 Pathogenic mechanisms of metabolic dysfunction–associated kidney disease (MDAKD): an overview

### 2.1 Metabolic functions of the kidney

The kidney functions not only as an excretory organ but also as a central metabolic organ, performing a range of essential physiological roles. These include the clearance of endogenous metabolites and exogenous toxins, involvement in drug metabolism, and the maintenance of water-electrolyte and acid-base homeostasis (Su et al., 2020). Additionally, the kidney regulates blood pressure, supports hematopoiesis, and modulates calcium-phosphate metabolism through the secretion of various hormones (Levassort and Essig, 2024),Such functions depend on the intricate coordination between diverse renal cell types and nephron structures (Peti-Peterdi et al., 2009).

At the systemic metabolic level, metabolic pathways play a pivotal role in the pathogenesis of kidney diseases. The kidney plays a central role in maintaining systemic energy homeostasis and metabolic equilibrium by regulating glucose, lipid, amino acid, and purine metabolic pathways. In terms of glucose metabolism, the kidney is the second most important gluconeogenic organ after the liver and contributes to blood glucose homeostasis through reabsorption in the proximal tubules (Gerich, 2010); Regarding lipid metabolism, glomerular and tubular epithelial cells are capable of uptaking, transporting, and oxidizing fatty acids to support energy supply, with approximately 90% of the energy required by renal tubular cells derived from fatty acid oxidation (Chang et al., 2021). Lipid metabolites further participate in cellular signaling and membrane integrity. The kidney also modulates purine metabolism by controlling uric acid production and excretion through transporters such as URAT1 and GLUT9, thus contributing to uric acid homeostasis (Mei et al., 2022).

Therefore, the kidney not only sustains systemic homeostasis through its excretory and endocrine functions but also serves as a central metabolic hub, coordinating energy conversion and nutrient sensing (Chrysopoulou and Rinschen, 2024). Its efficient metabolic processing and functional capacity are essential for preserving renal function as well as maintaining energy and metabolic homeostasis.

### 2.2 Metabolic dysregulation–induced renal injury

#### 2.2.1 Glucose dysregulation and AGEs accumulation

Glucose homeostasis is maintained through multiple coordinated mechanisms, including gastrointestinal absorption, uptake by skeletal muscle and adipose tissue, hepatic and renal gluconeogenesis, and renal and hepatic glucose reabsorption and excretion (Triplitt, 2012). The kidney plays a central role in this process by releasing glucose into the circulation via gluconeogenesis, uptaking circulating glucose to fulfill its energy requirements, and reabsorbing filtered glucose through proximal tubular transport. Cellular glucose uptake depends on transporter proteins residing in the plasma membrane. Three major families of glucose transporters have been identified in humans: GLUT proteins encoded by SLC2A genes, sodium-dependent glucose transporters (SGLTs) encoded by SLC5A genes, and SWEET proteins encoded by SLC50A genes (Wright, 2013). In renal tissues, SGLT and GLUT proteins are co-expressed and function synergistically to mediate glucose transport and reabsorption.

In MDAKD, glucose metabolic dysregulation is a key pathological process, characterized by imbalances in glucose uptake, gluconeogenesis, and reabsorption, resulting in sustained hyperglycemia. Under high-glucose conditions, glucose undergoes non-enzymatic glycation reactions with proteins and lipids, accumulating and forming toxic and irreversible compounds—advanced glycation end products (AGEs) (Wu et al., 2021). AGEs are endogenously generated under normal metabolic conditions, but their production is markedly increased in pathological states such as DKD and chronic inflammation (Delgado-Andrade, 2016). Their homeostasis is mainly affected by impaired clearance and increased endogenous generation, leading to accumulation and imbalance in the body (Fotheringham et al., 2022). In addition, AGEs can also be obtained exogenously from the diet, particularly from high-fat and high-sugar foods (Ma et al., 2025). AGEs deposit in the glomerular basement membrane, podocytes, and proximal tubules; thus, the kidney is not only the major organ responsible for AGE metabolism and excretion but also one of the most vulnerable target organs to AGE-induced damage (Dozio et al., 2023). In patients with kidney disease, this metabolic and excretory function is significantly impaired. Studies show that in healthy individuals, renal excretion accounts for approximately 30% of total AGE intake, whereas in patients with renal impairment, the excretion rate is only 5% (Chaudhuri et al., 2018). One of the most widely studied AGEs is Nε-(carboxymethyl)lysine (CML),formed by the reaction of lysine residues in proteins with glucose. CML is a key marker of AGE accumulation and is associated with multiple diseases, particularly DKD (Yamagishi, 2011),and circulating AGE levels are positively correlated with DKD mortality (Koska et al., 2022).

Because the kidney plays a central role in AGE metabolism and clearance, it is particularly vulnerable to damage resulting from AGE accumulation. AGEs exert pathogenic effects primarily through receptor-dependent mechanisms, notably via binding to their principal receptor, RAGE (receptor for advanced glycation end products). RAGE is broadly expressed in multiple cell types, including macrophages, endothelial cells, renal interstitial cells, and glomerular podocytes (Yan et al., 2004). The interaction between AGEs and RAGE promotes intracellular reactive oxygen species (ROS) generation and activates various signaling pathways. This, in turn, triggers inflammatory responses, oxidative stress, and fibrosis, induces apoptosis, exacerbates renal injury, and accelerates disease progression (Pal and Bhadada, 2023). Therefore, hyperglycemia can translate glucose metabolic dysregulation into oxidative stress, inflammatory activation, and profibrotic signaling through the AGEs/RAGE axis. These downstream pathological events not only directly mediate renal injury but also interact with abnormalities in lipid and purine metabolism, collectively promoting MDAKD progression through shared mechanisms involving oxidative stress, chronic inflammation, and fibrosis.

#### 2.2.2 Lipid dysregulation and lipid droplet accumulation

Lipid metabolism is essential for sustaining renal energy homeostasis and proper cellular function (Mitrofanova et al., 2023; Lee et al., 2024b). In the kidney, lipid metabolism comprises four major processes: the uptake of circulating lipids, *de novo* lipid synthesis, fatty acid β-oxidation, and cholesterol efflux. Under physiological conditions, these processes work together to maintain renal metabolic function and energy homeostasis.

In the setting of obesity, insulin resistance, and metabolic disturbances involving glucose and lipid homeostasis, circulating free fatty acid levels are elevated, accompanied by increased renal lipid uptake and impaired fatty acid oxidation. This imbalance leads to excessive lipid accumulation within the glomeruli, podocytes, and tubulointerstitial compartments of the kidney. Excessive renal lipid accumulation arises not only from increased uptake of exogenous fatty acids but also from enhanced endogenous lipid synthesis (Rong et al., 2024). Fatty acid transport proteins, particularly fatty acid transport protein 2 (FATP2) and cluster of differentiation 36 (CD36), facilitate the uptake of long-chain fatty acids into renal cells. Among these, CD36 has emerged as a key mediator of renal lipotoxicity (Listenberger et al., 2003), and its upregulation in metabolic kidney diseases, especially DKD, is closely associated with increased lipid uptake, oxidative stress, mitochondrial dysfunction, and progressive renal fibrosis (Niu et al., 2023). Consistently, genetic deletion of CD36 has been shown to ameliorate DKD by restoring fatty acid oxidation and improving mitochondrial function. In parallel, activation of *de novo* lipogenesis (DNL) further contributes to renal lipid deposition. During this process, acetyl-CoA carboxylase (ACC) and fatty acid synthase (FASN) catalyze the conversion of acetyl-CoA into saturated fatty acids, while stearoyl-CoA desaturase 1 (SCD1) promotes the synthesis of monounsaturated fatty acids. Experimental studies have demonstrated that deletion of FASN or ACC2 alleviates renal lipid accumulation in DKD, whereas increased SCD1 expression facilitates excessive lipid droplet formation and exacerbates lipotoxic injury (Radhakrishnan et al., 2008). Collectively, dysregulation of both fatty acid uptake and lipid synthesis promotes ectopic lipid deposition within the kidney, thereby driving oxidative stress, inflammation, and fibrosis during MDAKD progression.

Under physiological metabolic conditions, lipid droplets maintain lipid homeostasis and supply energy, and also coordinate fatty acid mobilization and metabolism via interactions with mitochondria and the endoplasmic reticulum (ER) (Nguyen et al., 2017). However, in the setting of metabolic imbalance, excessive lipid accumulation induces lipid peroxidation, mitochondrial damage, and ER stress. These alterations subsequently trigger oxidative stress, inflammatory responses, and apoptosis. Chronic lipid overload is termed “lipotoxicity” (Lytrivi et al., 2020), which impairs cellular function and causes tissue injury. Abnormal lipid droplet accumulation not only mediates lipotoxic damage but also disrupts cellular signaling pathways, aggravates renal metabolic dysfunction, and accelerates kidney injury and fibrogenesis (Kang et al., 2015).

Lipids are essential for cellular membrane integrity, signal transduction, and energy metabolism (Yoon et al., 2021). In the kidney, the tubulointerstitial region constitutes the main site of lipid deposition. Excessive lipid accumulation impairs the function of mesangial cells, podocytes, and tubular cells, leading to nephron structural damage and renal injury progression (de Vries et al., 2014). Thus, lipid metabolic dysregulation, aberrant lipid uptake, and excessive lipid droplet accumulation represent key pathological processes in the onset and progression of MDAKD (Gai et al., 2019). As the disease advances, renal lipid levels continue to rise, establishing a vicious cycle that further aggravates renal pathology.

Renal lipid metabolism is tightly controlled by multiple mechanisms, with fatty acid synthesis and oxidation playing central roles (Kang et al., 2015). In MDAKD, lipid metabolic dysregulation is not merely characterized by excessive lipid accumulation but is intricately linked to mitochondrial dysfunction, oxidative stress, and inflammatory activation. Increased fatty acid uptake, together with upregulation of lipid transporters such as CD36, promotes the intracellular accumulation of fatty acids and lipotoxic metabolites within renal cells. Concurrently, impaired fatty acid oxidation results in energy deficiency and excessive mitochondrial ROS production, which further activates pro-inflammatory and profibrotic signaling pathways, including the NLRP3 inflammasome, NF-κB, and TGF-β1 (Wang et al., 2022c). These downstream pathological events converge on shared mechanisms involving oxidative stress, chronic inflammation, and fibrosis, thereby contributing to the progression of MDAKD.

#### 2.2.3 Dysregulation of purine and uric acid metabolism

Purines constitute the basic building blocks of nucleic acids and are acquired both from the diet and via endogenous synthesis. The liver serves as the primary site for purine synthesis. Initially, purine nucleotides are synthesized and subsequently metabolized to hypoxanthine. Hypoxanthine is then oxidized to xanthine by xanthine dehydrogenase (XDH), which is further converted to uric acid (Kimura et al., 2021).

Purine catabolism culminates in the production of uric acid. Approximately two-thirds of uric acid is excreted renally, with the remaining one-third eliminated via the intestine, underscoring the kidney’s pivotal role in uric acid clearance. Owing to its low plasma protein binding affinity, uric acid is extensively filtered by the glomerulus and is subject to active secretion and reabsorption in the proximal tubules (Wen et al., 2024). This renal handling of uric acid contributes to systemic uric acid homeostasis. Consequently, serum uric acid levels are governed by the coordinated balance between hepatic uric acid production, renal excretion, and intestinal excretion and reabsorption (Liu et al., 2024).

Serum uric acid homeostasis is largely governed by renal uric acid transporters that mediate both reabsorption and secretion. In the proximal tubules, uric acid transporter 1 (URAT1) and glucose transporter 9 (GLUT9) facilitate uric acid reabsorption, whereas ATP-binding cassette subfamily G member 2 (ABCG2) and organic anion transporters (OATs) mediate its secretion (Han et al., 2025). Additionally, four key transporters—SLC2A9, SLC22A12, SLC17A1, and ABCG2—collectively modulate serum uric acid levels. Specifically, SLC2A9 and SLC22A12 correspond to GLUT9 and URAT1 and regulate uric acid reabsorption, while ABCG2 functions in both the kidney and intestine to mediate uric acid excretion and transport (Nakayama et al., 2017). Together, the coordinated activity of these transporters governs systemic uric acid balance, influencing serum uric acid concentrations and renal excretory capacity.

Dysregulation of uric acid metabolism in MDAKD is characterized by increased production and reduced excretion. Impaired renal uric acid excretion is a major contributor to hyperuricemia. Abnormal uric acid metabolism contributes to renal injury through multiple mechanisms. Under hyperuricemic conditions, excess uric acid accumulates and is transported to the kidneys via the circulation. When renal excretory function is compromised, uric acid deposits in the renal tubules and tubulointerstitial space, leading to urate crystal formation. These crystals induce tubular injury, tubulointerstitial inflammation, and cellular damage, thereby impairing renal function (Du et al., 2024). The deposition of urate crystals not only results directly from hyperuricemia but also triggers renal immune responses and oxidative stress. Within renal cells, uric acid activates NADPH oxidase and xanthine oxidase (XO), generating excessive reactive oxygen species (ROS), which in turn cause mitochondrial dysfunction, DNA damage, and apoptosis. Furthermore, uric acid activates the NF-κB pathway, promoting the release of inflammatory cytokines such as IL-6 and TNF-α, thereby driving chronic renal inflammation, glomerulosclerosis, and tubulointerstitial fibrosis (Gherghina et al., 2022).

Therefore, uric acid metabolic dysregulation not only constitutes a key pathogenic basis for hyperuricemia and hyperuricemic nephropathy but also contributes to MDAKD progression through the induction of oxidative stress, inflammatory activation, and profibrotic responses. In pathological conditions such as DKD and HN, hyperuricemia frequently coexists with hyperglycemia and lipid metabolic abnormalities, facilitating extensive metabolic crosstalk and reciprocal amplification among glucose, lipid, and purine metabolic pathways, which collectively drive renal injury and disease progression.

#### 2.2.4 Crosstalk among glucose, lipid, and purine metabolic dysregulation

Beyond the individual abnormalities in glucose, lipid, and purine metabolism described above, extensive pathological interactions exist among these metabolic disturbances, resulting in reciprocal amplification of renal injury. Persistent hyperglycemia promotes AGE formation and activation of the AGEs/RAGE signaling axis, thereby inducing ROS production and inflammatory responses. Importantly, hyperglycemia can also link glucose metabolic dysregulation to lipid metabolic abnormalities through lipid uptake–related pathways. Previous studies have shown that high glucose promotes free fatty acid uptake and lipid deposition in renal tubular cells via activation of the PPARγ/CD36 pathway, indicating that hyperglycemic conditions not only induce glucotoxicity but also exacerbate renal lipid burden (Feng et al., 2017). In diabetic kidneys, CD36-mediated lipid dysregulation suppresses mitochondrial fatty acid oxidation and enhances mitochondrial ROS production, leading to activation of the NLRP3 inflammasome in renal tubular epithelial cells and subsequent kidney injury (Hou et al., 2021). Thus, hyperglycemia can connect glucotoxicity, lipid accumulation, oxidative stress, and inflammatory responses into a continuous pathogenic cascade through key nodes such as AGEs/RAGE and CD36.

Lipid metabolic abnormalities may further influence uric acid homeostasis. Adipose tissue is not merely an energy storage organ but also exhibits purine metabolic activity through xanthine oxidoreductase (XOR)-mediated uric acid production and secretion. In obese mice, adipose tissue–derived uric acid secretion is significantly increased and is accompanied by elevated serum uric acid levels, whereas administration of the XOR inhibitor febuxostat markedly reduces circulating uric acid concentrations (Tsushima et al., 2013). These findings suggest that lipid accumulation and adipose tissue dysfunction may link lipid metabolic dysregulation to hyperuricemia by enhancing local purine metabolism and uric acid production. Conversely, hyperuricemia is not simply a metabolic consequence but may further aggravate adipose tissue inflammation and insulin resistance. Experimental studies have demonstrated that uric acid reduction decreases MCP-1 expression and macrophage infiltration in adipose tissue while increasing adiponectin levels and improving insulin resistance in obese mice, indicating that uric acid abnormalities may further amplify inflammation and metabolic imbalance associated with lipid dysregulation. Hyperuricemia has also been implicated in the development of insulin resistance through oxidative stress, endothelial dysfunction, and chronic low-grade inflammation, thereby further aggravating glucose metabolic abnormalities (Baldwin et al., 2011).

Glucose metabolic abnormalities may also affect uric acid homeostasis through insulin-dependent mechanisms. Toyoki et al. demonstrated that insulin upregulates the renal urate reabsorption transporter URAT1 while downregulating the urate efflux transporter ABCG2, thereby enhancing uric acid reabsorption and reducing uric acid excretion. This finding provides mechanistic evidence linking glucose metabolism to uric acid homeostasis (Toyoki et al., 2017).

Collectively, glucose, lipid, and purine metabolic disturbances are interconnected through key regulatory nodes, including AGEs/RAGE, CD36, XOR, URAT1/ABCG2, and NLRP3. These interactions ultimately converge on shared pathogenic pathways involving inflammatory activation, mitochondrial oxidative stress, gut–kidney axis dysfunction, and tubulointerstitial fibrosis, thereby promoting progressive renal injury.

From the perspective of disease subtypes, DKD is primarily characterized by chronic hyperglycemia, insulin resistance, and AGE accumulation, with pathological injury predominantly involving hyperglycemia-induced damage to endothelial cells, podocytes, and renal tubular epithelial cells (Sinha and Nicholas, 2023). In contrast, ORG is more strongly associated with obesity-related hemodynamic alterations, increased lipid uptake, lipid droplet accumulation, lipotoxicity, and maladaptive podocyte responses (Martínez-Montoro et al., 2022). HN is mainly characterized by excessive uric acid production, impaired uric acid excretion, urate crystal deposition, and uric acid–associated inflammatory responses (Zhang et al., 2025b). Although these disease subtypes may mutually reinforce one another through metabolic crosstalk and ultimately converge on common downstream processes, including inflammation, oxidative stress, and fibrosis, the dominant metabolic disturbances and key therapeutic targets differ substantially among them.

**FIGURE 1:**
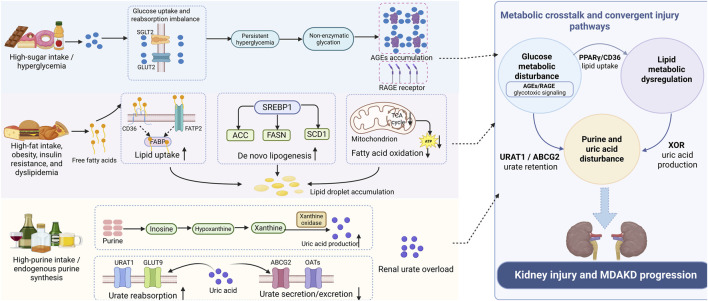
Metabolic dysfunction-driven kidney injury in metabolic dysfunction–associated kidney disease. Glucose, lipid, and purine/uric acid metabolic disturbances contribute to kidney injury through both independent and interconnected mechanisms. High-sugar intake and hyperglycemia induce glucose uptake and reabsorption imbalance involving SGLT2 and GLUT2, leading to persistent hyperglycemia, non-enzymatic glycation, AGEs accumulation, and RAGE activation. High-fat intake, obesity, insulin resistance, and dyslipidemia increase circulating free fatty acids, enhance renal lipid uptake through CD36, FATP2, and FABP, promote *de novo* lipogenesis through the SREBP1/ACC/FASN/SCD1 axis, and impair fatty acid oxidation, resulting in lipid droplet accumulation. High-purine intake and endogenous purine synthesis increase uric acid production through purine metabolism, while altered urate transport, characterized by increased URAT1/GLUT9-mediated reabsorption and decreased ABCG2/OATs-mediated secretion or excretion, contributes to renal urate overload. These metabolic abnormalities are further connected through crosstalk pathways, including AGEs/RAGE-mediated glucotoxic signaling, PPARγ/CD36-mediated lipid uptake, XOR-mediated uric acid production, and URAT1/ABCG2-mediated urate retention. Together, these processes converge on kidney injury and promote metabolic dysfunction–associated kidney disease (MDAKD) progression.

In summary, glucose metabolic dysregulation, lipid metabolic abnormalities, and purine/uric acid metabolic disorders are characterized by glucotoxicity, lipotoxicity, and uric acid overload, respectively. These metabolic disturbances interact and amplify one another throughout MDAKD progression, ultimately converging on common pathogenic mechanisms involving oxidative stress, inflammatory activation, mitochondrial dysfunction, and fibrosis, thereby leading to renal injury. An overview of the integrated mechanisms through which glucose, lipid, and purine metabolic dysregulation drive kidney injury is presented in Figure 1.

### 2.3 Activation of inflammatory factors and immune dysregulation

MDAKD, as a chronic progressive disease, is driven by persistent activation of inflammatory factors and immune dysregulation. Even in the absence of overt glucose and lipid metabolic disturbances, inflammation alone can independently promote renal structural and functional damage. In MDAKD, renal fibrosis is the most fundamental and prominent pathological feature, with inflammation playing a central role in its initiation and progression (Wada and Makino, 2013).

Local renal inflammatory cells can produce and secrete various pro-inflammatory and pro-fibrotic factors, which directly damage kidney structures and trigger epithelial-to-mesenchymal transition (EMT) in tubular epithelial cells (Liu, 2011). Patients with MDAKD often show elevated levels of pro-inflammatory cytokines, including TNF-α, IL-1β, IL-6, and MCP-1, both systemically and locally. These cytokines activate signaling pathways such as NF-κB, JAK/STAT, and MAPK (Wada and Makino, 2013), leading to tubular epithelial injury, interstitial fibrosis, and progressive renal dysfunction. Chronic inflammation can also induce programmed cell death of renal structural cells, disrupting the homeostasis of the renal microenvironment. The kidney’s innate immune system forms the first line of defense against external threats. By clearing metabolic wastes and toxins, it helps maintain immune homeostasis, suppressing both local and systemic inflammatory responses. Thus, beyond its pathogenic role, the immune system also supports the maintenance of renal immune equilibrium in healthy kidneys (Foresto-Neto et al., 2024).

In immune responses, the intrarenal immune system mainly consists of dendritic cells (DCs), with macrophages and fibroblasts also present in the renal interstitium (Zeisberg and Kalluri, 2015). In MDAKD kidney tissue, significant infiltration of immune cells, including macrophages and T cells, is observed. Overactivation of M1 macrophages and Th1/Th17 cells is considered key to maintaining the pro-inflammatory state, while the relative reduction of anti-inflammatory phenotypes, such as M2 macrophages and Treg cells, reflects an imbalance in immune regulation (Tang and Yiu, 2020). Renal tubular epithelial cells (TECs) and podocytes possess intrinsic immune capabilities, expressing various innate immune receptors, including Toll-like receptors (TLRs), inflammasomes, and nucleotide sensors. These cells recognize damage-associated molecular patterns (DAMPs), such as ATP, DNA, RNA, and uric acid, leading to the release of pro-inflammatory cytokines and chemokines, which recruit inflammatory cells to the kidney (Kurts et al., 2013). Additionally, glomerular endothelial cells and mesangial cells contribute to inflammatory responses, further exacerbating local immune activation and tissue injury. MDAKD inflammation is often sterile in nature (Lin et al., 2012), typically triggered by endogenous danger signals such as hyperglycemia, uremic toxins, fatty acids, and AGEs, thereby amplifying the inflammatory cascade. Continuous release of inflammatory factors not only reflects immune dysregulation but also drives the ongoing deterioration of renal function.

### 2.4 Mitochondrial dysfunction and oxidative stress

The kidney is one of the most energy-demanding organs in the human body and ranks second only to the heart in mitochondrial density and oxygen consumption (Pagliarini et al., 2008; Bhargava and Schnellmann, 2017). It performs critical functions, including metabolic waste clearance, nutrient reabsorption, fluid balance maintenance, and blood pressure regulation. Mitochondria, as key organelles and the cell’s metabolic hub, are central to aerobic metabolism. They primarily generate ATP through oxidative phosphorylation, converting the energy stored in glucose and lipids into ATP to fuel various cellular activities. As a mitochondria-rich organ with high energy requirements, the kidney relies on mitochondria to maintain the function of renal tubular epithelial cells, as well as filtration and reabsorption processes. Disruption of mitochondrial structure and function is closely linked to the onset and progression of various kidney diseases (Zhang et al., 2021).

Oxidative stress mainly involves ROS and reactive nitrogen species (RNS). ROS are byproducts of ATP synthesis and are predominantly produced in mitochondria. ROS, including hydrogen peroxide, superoxide anion, and hydroxyl radicals, play a central role in the pathogenesis and progression of kidney diseases (Sakashita et al., 2021). Studies have shown that oxidative stress can impair the normal functions of multiple renal cell types, induce endothelial dysfunction, and accelerate renal fibrosis (Ratliff et al., 2016).

Mitochondrial dysfunction occurs in multiple forms of MDAKD and plays a critical role, particularly in the pathogenesis of DKD (Liu et al., 2017). Damaged mitochondria result in excessive ROS levels, leading to oxidative stress-related injury (Forrester et al., 2018). ROS-mediated pathways induce DNA damage, mitochondrial dysfunction, lipid peroxidation, and abnormal protein modifications, which impair glomerular cell function and further exacerbate renal inflammation and fibrosis (Ozbek, 2012). Pathological processes associated with mitochondrial dysfunction include mitochondrial DNA damage, respiratory chain defects, dysregulation of mitochondrial dynamics, impaired autophagy, and excessive mitochondria-derived ROS production triggered by oxidative stress (Zhao et al., 2024). Accordingly, renal fibrosis in MDAKD is closely linked to oxidative stress and mitochondrial dysfunction (Patera et al., 2024).

### 2.5 Gut microbiota dysbiosis and gut–kidney axis dysfunction

Gut microbiota dysbiosis is closely associated with metabolic disorders. Gut microbiota, the resident microbial community in the human gastrointestinal tract, includes species such as Bifidobacterium and *Lactobacillus*, which synthesize vitamins and nutrients essential for human growth and development. The stability of gut microbiota is crucial not only for digestive health but also for maintaining overall metabolic homeostasis (Fan and Pedersen, 2021). Consistent with this concept, accumulating evidence from other metabolic diseases, particularly metabolic-associated fatty liver disease (MAFLD), has demonstrated that functional foods and dietary supplements can improve metabolic homeostasis through modulation of gut microbial composition and function, further emphasizing the central role of gut microbiota in metabolic health (Chang et al., 2025). The gut–kidney axis refers to the bidirectional interaction between the gut microbiome and the kidneys, playing a particularly important role in the progression of MDAKD. Recent studies increasingly indicate that gut microbiota and its metabolites are pivotal in MDAKD pathogenesis via the gut–kidney axis. Dysbiosis is defined as abnormal alterations in the composition and function of the gut microbial community, as well as disruption of intestinal barrier integrity (Evenepoel et al., 2017). Maintaining a balanced symbiosis between gut microbiota and the host is essential for health. By sustaining an appropriate balance between beneficial and potentially pathogenic bacteria, gut microbiota contributes to optimized metabolic function and reduces the risk of metabolic disorder-related diseases (Régnier et al., 2021).

Bioactive metabolites produced by gut microbiota during metabolism can be absorbed from the gut into the bloodstream, enter systemic circulation, and act as signaling ligands to regulate downstream pathways, thereby influencing multiple metabolic processes and organ functions (Holmes et al., 2020). Emerging evidence suggests that gut microbiota dysbiosis is accompanied by profound metabolic reprogramming, involving alterations in lipid, glucose, amino acid, and uric acid metabolism. Notably, gut microbiota metabolic reprogramming is increasingly recognized not merely as a consequence of metabolic disorders, but as an early pathogenic event that may actively drive disease initiation and progression through the remodeling of host metabolic and immune homeostasis (Wang et al., 2026).

Dysbiosis of the gut microbiota is closely linked to metabolic disorders (Chen et al., 2024; Zhang et al., 2015)and contributes to the development and progression of various types of MDAKD. Substantial clinical evidence also indicates that gut microbiota dysbiosis significantly accelerates the progression of CKD (Wong et al., 2014; Xu et al., 2017; Wang et al., 2023a). It also promotes the generation of gut-derived uremic toxins (GDUTs) (Lymperopoulos et al., 2022). In DKD, uremic toxins such as indoxyl sulfate, p-cresyl sulfate, and trimethylamine N-oxide (TMAO) accumulate abnormally when renal clearance is impaired, inducing oxidative stress, inflammation, and fibrosis, thereby accelerating further deterioration of kidney structure and function. Conversely, renal dysfunction leads to retention of metabolic products, including uric acid and oxalate, which further damage renal tissue, disrupt gut microbiota homeostasis, impair barrier function, and create a pro-inflammatory feedback loop that accelerates kidney disease progression (Chen et al., 2022). Disruption of intestinal barrier integrity increases intestinal permeability, facilitating the translocation of lipopolysaccharide (LPS), microbial metabolites, and other gut-derived toxins into the systemic circulation (Anders et al., 2013; Tsuji et al., 2024). The accumulation of these circulating factors contributes to systemic inflammation, oxidative stress, and immune dysregulation, thereby aggravating renal injury and promoting the progression of MDAKD.

In metabolic disorders, pathological alterations in the gut microbiome can compromise intestinal barrier integrity, allowing bacteria to translocate from the gut to extraintestinal organs such as the kidney. This process induces metabolic dysregulation, promotes the accumulation of uremic toxins, and worsens renal failure (Zhao et al., 2023a). Consequently, kidney diseases are often accompanied by gut microbiota dysbiosis and metabolic abnormalities, and these microbiota and metabolic changes further drive disease progression, creating a vicious cycle.

### 2.6 Tubulointerstitial injury and fibrosis progression

Tubulointerstitial injury and fibrosis are key pathological processes in the pathogenesis of MDAKD. The tubulointerstitial region is both a target of multiple pathological stimuli and a major determinant of renal function decline. Tubulointerstitial injury (TII) is an early and critical event in MDAKD development, reflecting the combined pathogenic effects of multiple factors. It is a progressive process from functional impairment to structural damage, involving cellular injury, inflammation, vascular abnormalities, and interstitial remodeling. Processes such as increased oxidative stress, inflammatory activation, tissue hypoxia, and fibrosis promotion can accelerate MDAKD progression (Wang et al., 2022a). These injuries not only disrupt intercellular communication and local homeostasis but also impair the renal microenvironment. In MDAKD patients, the degree of renal function loss is closely associated with the severity of tubulointerstitial injury and fibrosis (Wang et al., 2023b).

Fibrosis is a common pathological process triggered by abnormal fibroblast proliferation and excessive accumulation of extracellular matrix (ECM) due to multiple pathological factors (Ediga et al., 2023). It is characterized by abnormal growth of fibrous connective tissue and loss of parenchymal cells, leading to structural disruption and functional decline, and in severe cases, organ failure (Zhang et al., 2024a). Tubulointerstitial fibrosis (TIF) is not only a central pathological feature of chronic kidney disease but also one of the most reliable predictors of renal prognosis (Gewin, 2018). TIF involves nearly all kidney cell types, including fibroblasts, renal tubular epithelial cells, endothelial cells, mesangial cells, podocytes, and infiltrating immune cells such as lymphocytes and macrophages, each playing critical roles at different stages of this complex process. Key cellular events in TIF include inflammatory cell recruitment, fibroblast activation and proliferation, excessive ECM synthesis and deposition, and associated tubular atrophy and capillary rarefaction (Liu, 2011). Myofibroblasts and their precursor cells are considered the primary producers of ECM (Iwano et al., 2002). Together, these alterations lead to irreversible damage to renal structure and function.

In summary, the development and progression of MDAKD is a complex, multifactorial process involving multiple interconnected and reinforcing pathological mechanisms. These include the accumulation of toxic metabolites due to metabolic disorders, oxidative stress, inflammatory and immune activation, gut microbiota dysbiosis, and tubulointerstitial fibrosis, which interact and amplify one another to form a complex pathological network. Under metabolic abnormalities such as hyperglycemia, hyperlipidemia, and hyperuricemia, advanced glycation end-products (AGEs) and uremic toxins accumulate, inducing oxidative stress and impairing mitochondrial function, which in turn leads to excessive ROS production and disturbed energy metabolism (Forbes and Thorburn, 2018). Oxidative stress also activates pro-inflammatory pathways, resulting in immune imbalance and renal tubular epithelial cell injury (Nowotny et al., 2015). Meanwhile, gut microbiota dysbiosis and its metabolites, such as TMAO and indoxyl sulfate, exacerbate inflammation and fibrosis through the gut–kidney axis (Cao et al., 2022; Summers and Quimby, 2024). Collectively, these pathological factors converge on the tubulointerstitial region, disrupting intercellular communication, causing capillary rarefaction, and promoting abnormal extracellular matrix deposition, ultimately driving irreversible renal fibrosis.

**FIGURE 2:**
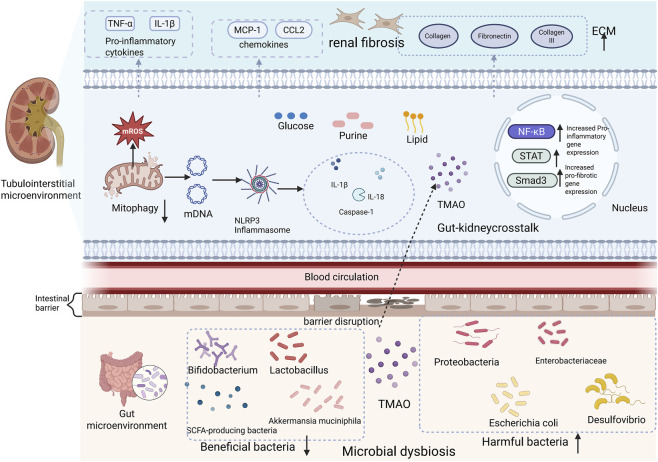
Multidimensional Mechanistic Overview of Natural Products in Modulating Metabolic-Associated Kidney Disease. This schematic illustrates the complex pathophysiology of metabolic-associated kidney diseases (MAKD), including diabetic kidney disease, obesity-related nephropathy, and hyperuricemia-induced renal injury, and highlights key intervention points of natural products. Metabolic stressors such as hyperglycemia, dyslipidemia, and purine accumulation induce oxidative stress, mitochondrial dysfunction, and mitophagy dysregulation, which activate NLRP3 inflammasome and downstream pro-inflammatory pathways (NF-κB, STAT, Smad3). Chronic inflammation, chemokine signaling (CCL2/MCP-1), and profibrotic gene expression (collagen III, fibronectin) contribute to tubulointerstitial injury and renal fibrosis. Gut dysbiosis, characterized by shifts in beneficial (Bifidobacterium, *Lactobacillus*, SCFA-producing bacteria, Akkermansia muciniphila) and harmful bacteria (Proteobacteria, Enterobacteriaceae, *Escherichia coli*, Desulfovibrio), exacerbates disease via gut–kidney crosstalk, increased TMAO production, and intestinal barrier disruption. Natural products—including polysaccharides, flavonoids, phenolic acids, triterpenoids, alkaloids, and saponins—target these multidimensional mechanisms, offering antioxidative, anti-inflammatory, immunomodulatory, metabolic regulatory, and anti-fibrotic effects.

Therefore, elucidating the multi-mechanistic pathological processes in MDAKD is crucial for a comprehensive understanding of the disease’s nature and progression. In this context, single-target therapies are often insufficient to effectively halt disease advancement. Consequently, simultaneous modulation of multiple key pathological pathways has become a critical focus for optimizing MDAKD treatment strategies. Natural products, with their diverse components and complex structures, often have the potential to target multiple pathways concurrently, allowing systemic modulation of metabolic regulation, antioxidation, anti-inflammation, immune homeostasis, and antifibrotic processes. Multidimensional mechanisms of natural products in modulating MDAKD, as illustrated in Figure 2.

## 3 Multidimensional mechanisms of natural products in modulating MDAKD

MDAKD is driven by multiple intertwined pathological mechanisms, involving oxidative stress, chronic inflammation, immune dysregulation, mitochondrial dysfunction, gut–kidney axis disturbance, and tubulointerstitial fibrosis. This complex pathological network not only poses challenges for MDAKD prevention and treatment but also highlights the importance of multi-target synergistic intervention strategies. Notably, natural products, as multifunctional small molecules, exhibit unique advantages in multiple pathological processes. Compared with single-target therapeutic strategies, natural products, owing to their structural diversity and bioactive regulatory capacity, show remarkable advantages in “multi-mechanism, multi-target” intervention. They can synergistically delay MDAKD progression through regulating oxidative stress responses, inhibiting inflammatory mediator release (Moudgil and Venkatesha, 2022), repairing mitochondrial function (Boeing et al., 2023), reshaping gut microbiota (Zhu et al., 2023), and suppressing fibrosis progression (Li et al., 2023). The following sections will systematically summarize the regulatory effects of natural products on the main pathological mechanisms and illustrate, through examples of representative bioactive compounds, their synergistic modulation and therapeutic potential in DKD, HN, and ORG.

### 3.1 Natural products targeting metabolic dysregulation in MDAKD

#### 3.1.1 Polysaccharides

Natural polysaccharides are macromolecules with broad bioactivities, including antitumor, antidiabetic, antioxidant, antiviral, and immunomodulatory effects (Zhao et al., 2023b), and they are particularly effective in regulating glucose and lipid metabolism (Xu et al., 2025). For instance, mulberry leaf polysaccharide (MLP), an active component from mulberry leaves, exerts notable hypoglycemic and lipid-lowering effects. MLP (100–400 mg/kg/day, administered orally for 10 weeks) has been shown to regulate bile acid metabolism by upregulating Cyp7a1, Cyp8b1, and TGR5 expression while inhibiting FXR, thereby improving glucose and lipid homeostasis and alleviating insulin resistance (Dai et al., 2024).

Polysaccharides also play an important role in modulating dysregulated glucose and lipid metabolism in MDAKD. Fucoidan (FPS), a sulfated cell-wall polysaccharide widely present in brown algae (Bilan and Usov, 2008), has demonstrated significant renal protective effects in streptozotocin (STZ)-induced DKD models (75–300 mg/kg/day for 10 weeks) (Wang et al., 2014), likely by improving glucose and lipid metabolism and lowering blood glucose.

The tricarboxylic acid (TCA) cycle, a key pathway in glucose and lipid metabolism, participates in the oxidation of carbohydrates, lipids, and amino acids, maintaining energy homeostasis (Akram, 2014). Ganoderma lucidum polysaccharide F31 (F31) has shown renal protective effects in db/db mice. Mechanistically, F31 (25–100 mg/kg/day for 10 days) increases ornithine levels, regulating the ornithine cycle, supporting nitrogen metabolism and TCA cycle activity. These effects collectively improve glucose metabolism, enhance insulin sensitivity, suppress gluconeogenesis, and alleviate DKD-related renal injury (Jiao et al., 2023).

#### 3.1.2 Flavonoids

Flavonoids are a class of polyphenolic compounds widely distributed in plants, characterized by a C6-C3-C6 carbon backbone. Their structural diversity confers multiple biological activities, including antioxidant, anti-inflammatory, and glucose- and lipid-regulatory effects, making them promising agents for the management of chronic metabolic diseases (Salaritabar et al., 2017). Based on the oxidation state of the C ring and the position of the B ring, flavonoids are mainly classified into flavones, flavonols, flavanones, flavanols (catechins), isoflavones, and anthocyanins (Panche et al., 2016).

In ORG, dysregulated lipid metabolism is a key pathogenic factor (Szeto et al., 2016). Cyanidin-3-glucoside (C3G) (10 mg/kg/day for 8 weeks) significantly alleviates lipid droplet accumulation in proximal tubular cells via inhibition of the PPARγ/CD36 pathway, corrects phospholipid metabolism, and reduces tubular injury and proteinuria, thereby conferring renal protection (Lu et al., 2025).

Targeting dysregulated purine–uric acid metabolism, apigenin 7-O-glucoside (AG) from Paeonia × suffruticosa exhibits multifaceted effects (Zhang et al., 2023b). AG (0.09–0.36 mg/kg/day for 4 weeks) inhibits xanthine oxidase to block uric acid synthesis and modulates renal urate transport by suppressing URAT1/GLUT9-mediated reabsorption while promoting OAT1/ABCG2-mediated secretion, enhancing uric acid clearance. This dual regulation of synthesis and excretion effectively reduces uric acid burden and mitigates HN-related renal pathology in hyperuricemic models.

#### 3.1.3 Phenolic acids

Flavonoids and phenolic acids are common polyphenolic natural products in plants, both exhibiting antioxidant and anti-inflammatory activities. Polyphenols can be classified into several structural types (Ajila et al., 2011), including phenolic acids (e.g., gallic acid, ferulic acid, caffeic acid), flavonoids (e.g., quercetin, catechin, epicatechin), anthocyanins, tannins, and lignins, all of which display broad biological activities. Flavonoids are characterized by a tricyclic skeleton and high structural diversity, whereas phenolic acids are based on a single benzene ring and can be further categorized into hydroxybenzoic acids and hydroxycinnamic acids, such as chlorogenic acid, ferulic acid, and caffeic acid (Shahidi and Yeo, 2018; Baptista et al., 2024). Chlorogenic acid (CGA), also known as 5-caffeoylquinic acid (5-CQA), is a widely distributed phenolic acid found in coffee, fruits and vegetables, and certain traditional Chinese medicines (Ferrare et al., 2018) ]. CGA has been shown to alleviate DKD progression by regulating lipid metabolism. In high-glucose and high-fat-induced cell and animal models, CGA (20–80 μM *in vitro* and 50 mg/kg/day for 8 weeks *in vivo*) reduces renal lipid accumulation, decreases the expression of fibrosis markers (α-SMA, FN, p-Smad3), and modulates lipid metabolism-related proteins by upregulating CPT1A and downregulating SREBP1c(Yang et al., 2024). These findings suggest that CGA ameliorates lipid metabolic disorders in renal tubular epithelial cells via inhibition of the Notch1/STAT3 signaling pathway.

### 3.2 Anti-inflammatory and immunoregulatory effects of natural products in MDAKD

Although MDAKD has traditionally been classified as a non-immune disease, numerous studies have shown that immune responses and inflammatory processes also play a key role in its onset and progression (Nguyen et al., 2006). Increasing evidence indicates that immune-mediated inflammatory processes are important contributors to the pathophysiology of MDAKD, suggesting that inhibition of chronic inflammation may help prevent and slow the development of renal fibrosis in MDAKD (Miguel et al., 2025).

#### 3.2.1 Polysaccharides

Ganoderma lucidum polysaccharides (GLP) exert significant renoprotective effects in DKD, primarily through anti-inflammatory mechanisms. Hu et al. (2022) reported that GLP (300 mg/kg/day for 10 weeks) treatment markedly reduced renal IL-6, IL-1β, and TNF-α levels in DKD rats. This effect may involve regulation of the PI3K/Akt/mTOR signaling pathway, thereby mitigating chronic inflammation and delaying MDAKD progression. Liriope radix polysaccharides (PSLR) demonstrated substantial renal protective effects in DKD models (Lu et al., 2014). PSLR (200–300 mg/kg/day for 8 weeks) upregulated nephrin and podocin expression, ameliorating renal dysfunction, proteinuria, and glomerular pathology in STZ-induced rats. The mechanism may involve inhibition of macrophage infiltration, reduction of T cell aggregation, and downregulation of NF-κB and p38 MAPK pathway-associated inflammatory mediators. Holothuria leucospilota polysaccharide (HLP) effectively improved renal function and pathological injury in DKD rats (Wen et al., 2025). HLP (100–200 mg/kg/day for 4 weeks) inhibited inflammatory mediators including MIF, CD74, and IL-6, modulated ERK1/2 and p38 signaling pathways, and activated the PI3K/AKT pathway to ameliorate podocyte injury, demonstrating renoprotective potential. Ramulus mori polysaccharides (RMP) (600 mg/kg/day for 30 days) ameliorated renal dysfunction and pathological injury in STZ-induced DKD mice (Guo et al., 2013). Mechanistically, RMP reduced renal levels of IL-6, IFN-γ, and TNF-α and suppressed the IL-1/NF-κB signaling pathway through downregulation of IL-1, IL-1R, p-IκB, and NF-κB expression, thereby attenuating renal inflammation.

#### 3.2.2 Flavonoids

AGEs have been identified as a major cause of HN, promoting inflammatory responses and oxidative cellular damage (Wang et al., 2013). Fisetin (3,3′,4′,7-tetrahydroxyflavone, FIS) is a natural dietary flavonoid. In a high-fat diet (HFD)-induced mouse model of obesity-related kidney disease, FIS (20–80 mg/kg/day for 16 weeks) inhibited the iRhom2/NF-κB signaling pathway, leading to reduced expression of inflammatory cytokines, including TNF-α, IL-6, IL-1β, IL-18, and CCL2. Moreover, FIS decreased macrophage infiltration (F4/80, CD68), thereby alleviating renal inflammation and improving kidney function (Chenxu et al., 2021).

Phyllanthi Fructus, a medicinal and edible natural fruit, is rich in flavonoids. These compounds (0.81 g/kg/day for 4 weeks) promote uric acid excretion by inhibiting xanthine oxidase activity, upregulating OAT1 and ABCG2, and downregulating URAT1. They also enhance superoxide dismutase activity, reduce MDA levels, and inhibit the expression of inflammatory cytokines, including IL-1β, IL-6, TNF-α, and NF-κB, thus ameliorating metabolic inflammation in hyperuricemic rats (Liang et al., 2024). Albanol A, a Diels–Alder active component from Morus alba L. root bark and a flavonoid-derived adduct, exerts notable anti-inflammatory and metabolic regulatory effects in hyperuricemia-associated kidney injury. It restores uric acid homeostasis by inhibiting xanthine oxidase and modulating multiple targets, including URAT1, GLUT9, and ABCG2. Additionally, Albanol A markedly suppresses the overexpression of IL-6, IL-1β, and TNF-α in the kidney, thereby alleviating hyperuricemia-induced renal inflammation and structural damage following MAR-EA treatment (100–400 mg/kg/day, 2 weeks) (Wang et al., 2025).

#### 3.2.3 Triterpenoids

Triterpenoids are a class of natural products synthesized by plants via the isoprenoid pathway, characterized by structural diversity and multiple biological activities. Recently, increasing attention has been paid to elucidating the regulatory mechanisms of triterpenoids and their roles in metabolic modulation (Dong and Qi, 2025). Oleanolic acid (OA) is a natural triterpenoid with antioxidant, anti-inflammatory, and hypoglycemic effects. Liu et al. (2022c) reported that in a high-fat diet (HFD) combined with STZ-induced DKD rat model, OA (50–100 mg/kg/day for 8 weeks) activated the AMPK/PGC-1α signaling pathway to enhance energy metabolism and inhibited the TLR4/NF-κB pathway to attenuate inflammation. OA also reduced macrophage infiltration and downregulated fibrosis-related proteins, including TGF-β1 and type IV collagen, thus conferring anti-inflammatory and renoprotective effects. Glycyrrhizin (GLC), a diol triterpenoid extracted from Glycyrrhiza uralensis, exhibits potent anti-inflammatory activity (Ogiku et al., 2011). Studies have shown (Thakur et al., 2017) that GLC (50 mg/kg, i. p., 5 days/week for 4 weeks) mitigates DKD-associated inflammation and renal injury by inhibiting high glucose-induced activation of HMGB1, TLR4, and NF-κB. Both *in vitro* and *in vivo* experiments indicate that GLC suppresses the release of pro-inflammatory cytokines and NLRP3 inflammasome activity, ultimately protecting renal function.

### 3.3 Natural products ameliorate oxidative stress and mitochondrial damage in MDAKD

#### 3.3.1 Polysaccharides

A novel heteropolysaccharide from Paeonia suffruticosa (MC-Pa) exhibits potent antioxidant activity. Lian et al. (2021) demonstrated through *in vitro* and *in vivo* pharmacological experiments that MC-Pa (64.5 μg/mL *in vitro*; 80–160 mg/kg/day for 12 weeks *in vivo*) enhances antioxidant defense systems and inhibits AGEs-induced ROS generation, thereby reducing oxidative injury. Consequently, MC-Pa alleviates mesangial expansion and renal tubular pathology in DKD rats, indicating its potential protective and therapeutic effects against MDAKD. High glucose-induced oxidative stress and mitochondrial dysfunction are critical factors contributing to podocyte insulin resistance and MDAKD progression.

#### 3.3.2 Flavonoids

Anthocyanins are highly hydroxylated flavonoids, characterized by a fully unsaturated C-ring and a hydroxyl group at the 3-position, serving as potential antioxidant therapeutics (Diaconeasa et al., 2015). Koh et al. (2015) reported that Seoritae extract (SE) (10 mg/kg/day for 12 weeks), which is rich in anthocyanins, activated the AMPK signaling pathway in db/db mice with DKD. This activation significantly reduced oxidative stress and lipotoxicity. SE also decreased proteinuria, mitigated renal lipid accumulation and apoptosis, and inhibited high glucose-induced oxidative damage, demonstrating substantial renoprotective effects.

Black rice bran extract (BRE) (100–200 mg/kg/day for 8 weeks), abundant in anthocyanin flavonoids, potently enhanced renal antioxidant defense by inhibiting the PKCα/Keap1 pathway and activating Nrf2 along with downstream antioxidant enzymes, including HO-1 and GCLC. BRE reduced lipid peroxidation products such as MDA and increased GSH levels, thereby alleviating oxidative stress and renal injury in high-fat diet-induced obesity-related kidney disease (Laorodphun et al., 2021).

#### 3.3.3 Phenolic acids

Gallic acid (GA) is a natural phenolic acid antioxidant, widely distributed in plants such as bearberry leaves, pomegranate root bark, and gallnuts, and exhibits notable effects on metabolic regulation (Garud and Kulkarni, 2018). Studies have shown Lee et al. (2024a) that in HFD-fed db/db mice, GA (100 mg/kg for 12 weeks) downregulated miR-709a-5p, relieving its inhibition of the antioxidant transcription factor NFE2L2. This enhanced the activity of antioxidant enzymes, including catalase, glutathione peroxidase, and superoxide dismutase, thereby significantly reducing renal oxidative stress in DKD. Another study (Mojadami et al., 2023) demonstrated that GA (30 mg/kg/day, p. o.) effectively ameliorated methylglyoxal (MG)-induced DKD. GA upregulated Nrf2 and antioxidant enzyme activities (SOD, CAT, Glo1), inhibited oxidative stress, and modulated fibrosis- and ER stress-associated miRNAs (downregulating miR-192 and miR-204, upregulating miR-29a), resulting in improved renal function and reduced histopathological damage. These findings suggest that GA, as a natural antioxidant, may have potential as a supportive therapeutic approach for MDAKD.

Grape pomace extract (GPE), rich in phenolic compounds, exhibits potent antioxidant activity. *In vitro* and *in vivo* studies showed that GPE (300 mg/kg body weight/day for 16 weeks) markedly reduced reactive oxygen species generation in the kidneys of obesity-related kidney disease models. It inhibited Nox4 expression, mitigated oxidative stress, and alleviated tubular and glomerular damage, thereby protecting renal function (Figueras et al., 2025). Ferulic acid (FA), a common phenolic acid present in fruits and plants, also exerts renoprotective effects. Qi et al. (2020) reported that FA (100 mg/kg/day for 8 weeks) treatment in STZ-induced DKD rats significantly enhanced antioxidant enzyme activities (SOD, CAT, GPx), reduced renal MDA levels, and mitigated oxidative stress. FA also improved renal function parameters and histopathological injury, indicating its protective role via antioxidant mechanisms.

Ethanol extract of Chinese sumac fruit (CE) is a natural polyphenolic preparation derived from Rhus chinensis Mill. fruit. Studies indicated (Ma et al., 2024) that CE (400–800 mg/kg/day for 6 months) increased antioxidant enzyme activities (SOD, GSH) and decreased lipid peroxidation products (MDA), thereby alleviating hyperuricemia-induced renal oxidative stress and improving kidney injury associated with elevated uric acid levels.

### 3.4 Modulation of gut microbiota and restoration of the “gut-kidney axis” by natural products in MDAKD

#### 3.4.1 Polysaccharides

The gut microbiota not only participates in polysaccharide metabolism but is also profoundly modulated by bioactive polysaccharide components, conferring potential therapeutic benefits in various disease settings (Ho Do et al., 2021). Recent evidence suggests that natural products can exert therapeutic effects through multiple mechanisms, among which the nonabsorptive pathway has attracted increasing attention. Notably, many poorly absorbed components, particularly polysaccharides, can regulate gut microbiota composition, microbial metabolites, and intestinal barrier function. In MDAKD, this mode of action provides an important mechanistic basis for the renoprotective effects of natural products through modulation of the gut–kidney axis (Zhai et al., 2025). Bupleurum polysaccharides have been demonstrated to exert renoprotective effects in DKD, likely via gut microbiota modulation. Studies (Feng et al., 2019) showed that Bupleurum polysaccharides (60 mg/kg/day for 6 weeks) markedly improved glycemic control and renal function in STZ-induced DKD mice, restored gut microbiota diversity, enhanced intestinal barrier integrity, and ameliorated kidney injury, suggesting therapeutic potential through regulation of the gut microbiota and repair of the“gut-kidney axis”. Similarly, Cordyceps cicadae polysaccharides (CCP) (75–300 mg/kg/day for 4 weeks)conferred beneficial effects in DKD rats by improving insulin resistance and glucose tolerance, while reshaping gut microbiota composition, particularly increasing the abundance of beneficial bacteria, thereby alleviating “gut-kidney axis” dysfunction (Yang et al., 2020).

BXP (100–300 mg/kg/day for 15 days), a polysaccharide isolated from Dioscorea septemloba, has been shown to reduce serum uric acid levels in a hyperuricemia (HUA) mouse model by inhibiting xanthine oxidase (XOD) activity and modulating renal urate transporters (GLUT9, URAT1, OAT1, OAT3, ABCG2), thereby alleviating kidney and liver injury. Additionally, BXP regulates gut microbiota composition and promotes short-chain fatty acid (SCFA) production, with its renoprotective effects primarily mediated via the gut–kidney axis (Wang et al., 2024). Corn silk, a medicinal and edible plant, contains corn silk polysaccharides (CSP) as its main active component. Early CSP (100–400 mg/kg/day for 8 weeks) intervention has been shown to effectively reshape gut microbiota composition and metabolic profiles in DKD rats (Dong et al., 2023). Key metabolic pathways affected include fatty acid and bile acid metabolism, and major microbial taxa modulated include Firmicutes, Bacteroidetes, the NK4A136 group of Lachnospiraceae, and Dubosiella. These findings suggest that CSP exerts therapeutic effects by regulating the “gut microbiota–metabolism–kidney” axis.

#### 3.4.2 Flavonoids

Flavonoids from Opuntia ficus-indica fruit (OFI-F) exhibit potent pharmacological activities, including gut microbiota modulation and anti-diabetic effects (Martins et al., 2023). High-purity OFI-F (300–500 mg/kg/day for 3 weeks) has been demonstrated to regulate gut microbiota composition, increase short-chain fatty acid (SCFA) production, restore intestinal barrier integrity, and consequently improve renal function in DKD mice (Zhang et al., 2022). Green tea catechins (GTCs), a class of flavonoids, can modulate gut microbiota and ameliorate obesity-related kidney disease. *In vitro*, GTCs (150 or 300 mg/kg) reduced lipid droplet accumulation in fatty acid-treated renal tubular cells and regulated PPARγ and CD36 expression. *In vivo*, GTCs improved renal injury by modulating the PPARγ/CD36 signaling pathway, promoting beneficial bacteria such as Akkermansia muciniphila and *Lactobacillus* reuteri, and restoring gut-kidney axis function (Patial et al., 2023). Astragalus aqueous extract (AAE) ameliorates HN via the gut-kidney axis, as confirmed by multi-omics studies (Qu et al., 2025). Flavonoids in AAE (1.5–3.0 g/kg/day) modulate microbiota composition, increase the Firmicutes/Bacteroidetes ratio and *Lactobacillus* abundance, and enhance SCFA production, thus remodeling the purine metabolism network. They also downregulate key enzymes including XDH, MPO, and HPRT1, reducing uric acid burden and renal tubular injury.

#### 3.4.3 Phenolic acids

CGA, a phenolic acid widely found in plants, has been shown to prevent HN. In a high uric acid–induced rat model, CGA (40 mg/kg/day) was demonstrated to modulate the gut microbiota by reducing the abundance of bacteria involved in TMAO production, such as Blautia and *Enterococcus*. This modulation led to decreased TMAO levels, suppression of the PI3K/AKT/mTOR signaling pathway, and subsequent improvement in renal function and renal histopathology in HN rats (Zhou et al., 2022).

### 3.5 Antifibrotic effects of natural products and tubular protection in MDAKD

Tubulointerstitial fibrosis, a chronic and progressive pathological process driven by aging and/or CKD, is associated with sustained impairment and decline of renal function (Zhang et al., 2023a). Increasing evidence demonstrates that natural products and their bioactive constituents provide protective effects against renal fibrosis and preserve tubular structure and function in MDAKD.

#### 3.5.1 Polysaccharides

Polysaccharides are recognized as potential antifibrotic agents among natural products (Wang et al., 2022b). Dendrobium officinale polysaccharide (DOP), a major active constituent of Dendrobium officinale, exhibits glucose- and lipid-lowering effects. In studies using db/db mice, oral administration of DOP (400 mg/kg/day) significantly downregulated the highly expressed lncRNA XIST and TGF-β1 in renal tissues of DKD mice. XIST gene knockout further confirmed that DOP may mitigate renal fibrosis by modulating these signaling pathways, demonstrating its antifibrotic potential (Zhang et al., 2024c). Similarly, APS was found to regulate a novel lncRNA, Gm41268, exerting renal protective effects in DKD via the Gm41268/PRLR pathway. Specifically, APS (2 g/kg diet for 12 weeks) decreased the expression of Gm41268 and its target gene PRLR, activated autophagy, inhibited the mTOR signaling pathway, and reduced TGF-β, fibronectin, and type IV collagen levels, thereby attenuating renal fibrosis (Chen et al., 2023).

#### 3.5.2 Flavonoids

DCE (100–200 mg/kg/day *in vivo*; 10–25 μg/mL *in vitro*), rich in flavonoids and saponins, attenuates renal fibrosis by suppressing TGF-β1 signaling, blocking EMT progression, and reducing α-SMA, collagen I, and fibronectin expression. DCE also modulates ABCG2, OAT3, URAT1, and GLUT9, promoting uric acid excretion and inhibiting xanthine oxidase activity, thereby mitigating HUA-induced renal fibrosis (Lin et al., 2025).

MHE (25, 50, 100 mg/kg/day), containing abundant flavonoids, exhibits potent antifibrotic effects in a HUA-induced renal injury mouse model. It downregulates key fibrotic mediators including TGF-β1, α-SMA, and collagen I, reduces collagen deposition, and suppresses renal interstitial remodeling, effectively alleviating fibrosis in HUA-related kidney disease (Guo et al., 2025). EELC (250 and 500 mg/kg/day *in vivo*), rich in flavonoids, markedly inhibits renal JAK2/STAT3 signaling in adenine/potassium oxonate-induced HN mice, reducing fibrosis-related factors and interstitial remodeling, thereby exerting renal antifibrotic effects and improving kidney function (Pan et al., 2021).

SCU (25, 50, and 100 mg/kg/day *in vivo*), a flavonoid derived from Scutellaria baicalensis, has been reported by Yi et al. to attenuate renal fibrosis in obesity-associated kidney disease models by inhibiting AP-1 (FOS/JUN) signaling, downregulating TGF-β1 and fibronectin expression, and reducing collagen deposition and interstitial remodeling (Yi et al., 2025).

#### 3.5.3 Saponins

Astragaloside I (ASI), a bioactive saponin isolated from Astragalus, exhibits renoprotective effects in DKD. Zhang et al. (2024b) demonstrated that Astragaloside I (10–40 mg/kg/day *in vivo*; 5–20 μM *in vitro*) ameliorates renal fibrosis in DKD mice by inhibiting HDAC3 expression and activating Klotho. This intervention blocks the TGF-β1/Smad2/3 signaling pathway, leading to reduced expression of fibrotic mediators, including α-SMA and collagen. Notoginsenoside R1 (NGR1), a novel saponin derived from Panax notoginseng, activates the Nrf2/HO-1 pathway, suppresses TGF-β1–associated fibrosis, and alleviates ROS accumulation and apoptosis. Both *in vitro* and *in vivo* studies confirm that Notoginsenoside R1(30 mg/kg/day *in vivo*; 25 μM *in vitro*) effectively mitigates DKD-related renal injury and exerts pronounced antifibrotic effects (Zhang et al., 2019).

LS-102, a derivative of Astragaloside I, was evaluated in a high-fat diet (HFD)–induced ORG mouse model with varying doses of Astragaloside I and LS-102 (Li et al., 2022). LS-102 (10, 40 mg/kg/day) markedly alleviated renal fibrosis and improved kidney function by inhibiting M1 macrophage infiltration in perirenal adipose tissue, downregulating inflammatory mediators, and suppressing the TGF-β1/Smad signaling pathway, ultimately reducing the expression of fibrotic factors.

### 3.6 Integrated synergistic mechanism: Multi-target combined intervention in MDAKD progression

With the growing understanding of MDAKD pathogenesis, it has become increasingly evident that disease progression is not driven by a single pathogenic pathway, but rather by a complex and interconnected network involving metabolic dysregulation, oxidative stress, inflammation, gut microbiota dysbiosis, and renal fibrosis (Fan et al., 2025). These pathological processes are highly interdependent and mutually reinforcing, forming self-perpetuating pathogenic circuits that collectively accelerate kidney injury and disease progression. Consequently, therapeutic strategies targeting a single pathway may be insufficient to comprehensively interrupt the multifactorial pathogenesis of MDAKD. In contrast, natural products, owing to their multi-component and multi-target characteristics, have the potential to simultaneously modulate multiple interconnected pathogenic processes and exert coordinated regulatory effects across disease-associated networks. Importantly, although the available evidence is derived from different MDAKD subtypes, including diabetic kidney disease and hyperuricemic nephropathy, these studies consistently suggest that the therapeutic benefits of natural products may arise from their capacity to orchestrate multidimensional regulation of shared pathogenic mechanisms rather than isolated molecular targets.

Quercetin represents a typical example of the multidimensional regulatory potential of natural products in MDAKD. Beyond its lipid-lowering effects, quercetin simultaneously targets metabolic dysregulation and gut–kidney axis dysfunction. In DKD models, quercetin enhances fatty acid oxidation and restores lipid homeostasis through PPARα activation (Liu et al., 2022a; Guo et al., 2024; Hosseini et al., 2021). Meanwhile, in hyperuricemic nephropathy, it modulates gut microbiota composition and metabolism, reduces the production of gut-derived uremic toxins, and improves intestinal barrier integrity and gut–kidney axis homeostasis (Peng et al., 2025). These findings suggest that quercetin exerts coordinated renoprotective effects through the concurrent regulation of metabolic and microbiota-associated pathogenic pathways.

Compared with natural products that primarily target a limited number of pathogenic pathways, apigenin exhibits a broader spectrum of regulatory activities across multiple interconnected processes involved in MDAKD progression. Accumulating evidence suggests that apigenin simultaneously modulates metabolic abnormalities, inflammation, oxidative stress, and fibrosis. In experimental models, apigenin reduces serum uric acid levels and attenuates renal injury, while suppressing MAPK-mediated inflammatory signaling and decreasing the expression of TNF-α, IL-6, and NF-κB (Liu et al., 2022b; Malik et al., 2017). Furthermore, apigenin alleviates oxidative stress by inhibiting CD38, activating SIRT3, and improving mitochondrial function and antioxidant defenses (Ogura et al., 2020). It also exerts antifibrotic effects through inhibition of the TGF-β1/EMT axis and Wnt/β-catenin signaling pathways, thereby reducing collagen deposition and tubulointerstitial fibrosis (Li et al., 2021). Given the close interplay among these pathogenic processes, the ability of apigenin to coordinately regulate multiple disease-associated pathways may underlie its comprehensive renoprotective effects, highlighting its potential as a multitarget therapeutic agent for MDAKD.

The multidimensional regulatory capacity of natural products is further reflected in other bioactive compounds that simultaneously influence several interconnected pathogenic pathways. Astragalus polysaccharide (APS) modulates oxidative stress, inflammation, fibrosis, and gut microbiota homeostasis through regulation of the Nrf2, TLR4/NLRP3/NF-κB, and TGF-β/Smad signaling pathways (Guo et al., 2023). Likewise, berberine attenuates DKD progression by concurrently suppressing NLRP3 inflammasome activation and epithelial–mesenchymal transition (EMT), thereby mitigating inflammatory responses and renal fibrotic remodeling (Ma et al., 2022). These findings collectively indicate that the therapeutic effects of natural products are often achieved through coordinated regulation of multiple interconnected pathogenic processes rather than isolated molecular targets.

Taken together, the collective evidence highlights that the therapeutic value of natural products extends beyond modulation of individual molecular targets and is more accurately characterized by their capacity to orchestrate coordinated regulation across multiple interconnected pathogenic networks. Given the intricate crosstalk among metabolic dysregulation, inflammation, oxidative stress, gut microbiota dysbiosis, and fibrosis during MDAKD progression, simultaneous intervention in these processes may provide a mechanistically rational approach for disease management. Such a multidimensional mode of action not only distinguishes natural products from conventional single-target interventions but also underscores their potential as promising candidates for the development of network-based therapeutic strategies in MDAKD. Table 1 summarizes the multidimensional mechanisms by which natural products modulate MDAKD progression.

**TABLE 1:** Multidimensional mechanistic summary of natural products in modulating metabolic-associated kidney disease.

Mechanism	Category of Natural Products	Representative Compounds	Targets	Models	Effects	References
Regulation of metabolic dysregulation	Polysaccharides	Mulberry leaf polysaccharide (MLP)	Cyp7a1, Cyp8b1, TGR5, FXR; bile acid metabolism	STZ-induced DKD rats	Improves glucose and lipid metabolism and alleviates insulin resistance	Dai et al. (2024)
		Fucoidan (FPS)	Glucose–lipid metabolic pathways; TCA cycle; metabolic enzymes	STZ-induced DKD rats	Mitigate metabolic abnormalities; reduce blood glucose; improve renal function and morphometry	Bilan and Usov (2008)
		Ganoderma lucidum polysaccharide F31	Ornithine cycle; nitrogen metabolism; TCA cycle	db/db mice (DKD model)	Improves glucose metabolism; enhances insulin sensitivity; suppresses gluconeogenesis; alleviates DKD-related renal injury	Jiao et al. (2023)
	Flavonoids	Cyanidin-3-glucoside (C3G)	PPARγ/CD36 pathway; glycerophospholipid metabolism	ORG mice; proximal tubular cells	Improved lipid metabolism; reduced lipid deposition and proteinuria; alleviated tubular injury	Szeto et al. (2016)
		Apigenin 7-O-glucoside, AG	Xanthine oxidase; URAT1; GLUT9; OAT1; ABCG2	High purine diet–induced HN mice	Reduces serum uric acid and creatinine levels; suppresses oxidative stress; inhibits xanthine oxidase activity; modulates renal urate transporters; alleviates renal injury	Zhang et al. (2023b)
	Phenolic acids	Chlorogenic acid (CGA)	Notch1/STAT3 signaling; CPT1A/SREBP1c; lipid metabolism regulators	DKD mice; HK2 cells	Reduced renal lipid accumulation; decreased fibrosis markers (α-SMA, FN, p-Smad3); improved tubular lipid metabolism via Notch1/STAT3 inhibition	Ferrare et al. (2018)
Anti-inflammatory and immunoregulatory effects	Polysaccharides	Ganoderma lucidum polysaccharides (GLP)	PI3K/Akt/mTOR pathway	STZ-induced DKD rats	Reduces renal inflammatory cytokines and mitigates chronic inflammation in DKD	Hu et al. (2022)
		Liriope radix polysaccharides (PSLR)	NF-κB/p38 MAPK pathway	STZ-induced DKD rats	Reduced renal inflammation; improved glomerular structure; decreased proteinuria; enhanced nephrin and podocin expression	Lu et al. (2014)
​	​	Holothuria leucospilota polysaccharide (HLP)	ERK1/2, p38, PI3K/AKT pathways	GK rats (DKD model)	Reduced renal inflammation and fibrosis; improved podocyte function; enhanced renal filtration; alleviated kidney enlargement	Wen et al. (2025)
		Ramulus mori polysaccharides (RMP)	IL-1/NF-κB pathway	STZ-induced DKD rats	Reduced renal inflammation; restored cellular homeostasis; mitigated renal injury	Guo et al. (2013)
	Flavonoids	Phyllanthi fructus (YGZ)	Xanthine oxidase (XDH); renal urate transporters (URAT1, OAT1, ABCG2); NF-κB signaling	High-fat/high-sugar diet plus potassium oxonate/adenine–induced HN rats	Suppresses inflammation and immune activation; enhances urate excretion; alleviates renal injury	Liang et al. (2024)
		Fisetin (FIS)	iRhom2/NF-κB signaling; TNF-α, IL-6, IL-1β, IL-18, CCL2; F4/80, CD68	High-fat diet–induced ORG mice; PAL-stimulated macrophages	Suppresses inflammation and oxidative stress; ameliorates metabolic disorder–associated renal injury	Chenxu et al. (2021)
		Morus alba L. root bark (MAR); albanol A	XOD; URAT1; GLUT9; ABCG2; NF-κB	Hyperuricemia-induced HN mice	Inhibits uric acid synthesis; modulates renal urate transporters; suppresses renal inflammation; alleviates renal injury	Wang et al. (2025)
	Triterpenoids	Oleanolic acid (OA)	AMPK/PGC-1α, TLR4/NF-κB	HFD + STZ-induced DKD rats	Mitigate inflammation, improve lipid metabolism, reduce fibrosis, protect kidney function	Liu et al. (2022c)
		Glycyrrhizin (GLC)	HMGB1/TLR4/NF-κB pathway; NLRP3 inflammasome; pro-inflammatory cytokines	ZDF rats, NRK-52E cells (high glucose)	Reduce inflammation, suppress pro-inflammatory cytokines and NLRP3 activation, protect kidney function	Thakur et al. (2017)
Mitigate oxidative stress and mitochondrial damage	Polysaccharides	MC-Pa (polysaccharide from Moutan Cortex)	Oxidative stress, TGF-β1, AGEs-induced ROS	DKD rats; vascular endothelial cells	Reduced oxidative stress and ROS, alleviated glomerular and tubular damage	Lian et al. (2021)
	Flavonoids	Anthocyanin-rich Seoritae extract (SE)	AMPK signaling; oxidative stress; lipotoxicity	db/db mice, human glomerular endothelial cells	Reduced albuminuria, decreased renal lipid accumulation, inhibited apoptosis, alleviated oxidative stress, improved glomerular pathology	Koh et al. (2015)
		Black rice bran extract (BRE)	PKCα/Keap1/Nrf2 pathway; HO-1; GCLC; MDA; GSH	High-fat diet–induced ORG rats	Suppresses apoptosis and inflammation; improves metabolic disturbance; alleviates renal injury	Laorodphun et al. (2021)
​	Phenolic acids	Gallic Acid (GA)	miR-709a-5p → NFE2L2, antioxidant enzymes (catalase, GPx, SOD)	db/db mice on high-fat diet; MES-13 renal cells	Enhanced antioxidant defense, reduced renal oxidative stress and lipid accumulation, alleviated DKD	Lee et al. (2024a)
			Nrf2, antioxidant enzymes, fibrosis/ER stress–related miRNAs	Methylglyoxal-induced DKD in male mice	Enhanced antioxidant defense, reduced oxidative stress, improved kidney function, and alleviated renal tissue damage	Mojadami et al. (2023)
		Ferulic acid (FA)	SOD, CAT, GPx, MDA	STZ-induced DKD rats	Enhances antioxidant enzyme activities, reduces renal MDA levels, improves renal function, and alleviates histopathological injury	Qi et al. (2020)
		Grape pomace extract (GPE)	ROS; Nox4	HFD-induced ORG mice	Regulates energy metabolism; suppresses inflammasome activation and renal inflammation; alleviates lipotoxic renal injury	Figueras et al. (2025)
		Chinese sumac fruit extract (CE; Rhus chinensis Mill.)	SOD, GSH, MDA	High-purine yeast diet–induced HN mice	Lowers uric acid; enhances urate excretion; inhibits renal inflammation; alleviates renal injury	Ma et al. (2024)
Modulation of gut microbiota and restoration of the gut–kidney axis	Polysaccharides	Bupleurum polysaccharides	Gut microbiota diversity; gut barrier; kidney and colon inflammation	STZ-induced DKD mice	Improved glycemic control and renal function; restored microbiota diversity; reduced kidney and colon inflammation	Feng et al. (2019)
		Cordyceps cicadae polysaccharides (CCP)	Gut microbiota composition; beneficial bacteria	HFD + STZ-induced DKD rats	Improved glycemic control and renal function; restored microbiota diversity; reduced kidney and colon inflammation	Yang et al. (2020)
		Dioscorea septemloba polysaccharide (BXP)	XOD; URAT1; GLUT9; OAT1; OAT3; ABCG2	HUA-induced mice	Lowers uric acid; regulates urate transporters; modulates gut microbiota; attenuates renal fibrosis	Wang et al. (2024)
		Corn silk polysaccharides (CSP)	Gut microbiota–metabolite–kidney axis	STZ-induced DKD rats	Reshaped gut microbiota and metabolite profiles; improved metabolic homeostasis; alleviated renal inflammation and injury	Dong et al. (2023)
​	Flavonoids	Flavonoids from Opuntia ficus-indica fruit (OFI-F)	Modulation of gut microbiota, SCFAs production, intestinal barrier repair	DKD mice (HFD + STZ)	Improved renal function, alleviated hyperglycemia and renal injury, reduced inflammation, repaired gut–kidney axis	Zhang et al. (2022)
		Green tea catechins (GTCs)	PPARγ/CD36; gut microbiota	HFD-induced ORG mice	Modulates renal lipid uptake; improves insulin sensitivity; restores gut microbiota; attenuates obesity-induced kidney injury	Patial et al. (2023)
		Astragalus aqueous extract (AAE)	XDH, MPO, HPRT1	HN rats	Modulates gut-kidney axis; regulates purine metabolism; reduces renal inflammation; improves SCFA production and gut microbiota balance	Qu et al. (2025)
Anti-fibrotic effect and renal tubular protection	Phenolic acids	Chlorogenic Acid (CGA)	Gut microbiota (reduced Blautia and *Enterococcus*), TMAO production, PI3K/AKT/mTOR signaling	HN mice	Improved kidney function, reduced TMAO levels, alleviated fibrosis, restored gut microbiota balance	Zhou et al. (2022)
	Polysaccharides	Dendrobium officinale polysaccharide (DOP)	lncRNA XIST, TGF-β1	db/db mice (DKD model)	Reduced renal interstitial fibrosis; downregulated lncRNA XIST and TGF-β1; delayed DKD progression	Zhang et al. (2024c)
		Astragalus polysaccharide (APS)	lncRNA Gm41268, PRLR, mTOR, autophagy, TGF-β, FN, Collagen IV	db/db mice (DKD model); NRK-52E cells (high glucose)	Reduced fibrosis and improved kidney function	Chen et al. (2023)
	Flavonoids	Desmodium caudatum extract (DCE)	TGF-β1, Slug, E-cadherin, ABCG2, OAT3, URAT1, GLUT9	Adenine-induced HUA mice; UA-treated NRK52E cells	Suppresses TGF-β1-mediated EMT; modulates urate transporters; reduces renal fibrosis and uric acid–induced kidney injury; comparable reno-protection to allopurinol	Lin et al. (2025)
		Moslae Herba extract (MHE)	XOD, ABCG2, NLRP3, JAK2/STAT3	PO + HX–induced HN mice	Inhibits XOD; enhances urate excretion via ABCG2; suppresses NLRP3/JAK2-STAT3–mediated renal inflammation and fibrosis	Guo et al. (2025)
		Ethanol extract of Liriodendron chinense barks (EELC)	XOD; OAT1; OAT3; ABCG2; NF-κB; ASK1/JNK/c-Jun; JAK2/STAT3	Adenine/potassium oxonate–induced HN mice	Lowers serum uric acid; enhances urate excretion; suppresses renal inflammation and fibrosis; improves renal function	Pan et al. (2021)
​	​	Scutellarin (SCU)	AP-1 (FOS/JUN); TGF-β1; FN	HFD-induced ORG rats	Suppresses AP-1 activation; inhibits renal fibrosis; improves renal function	Yi et al. (2025)
	Saponins	Astragaloside I (ASI)	HDAC3 inhibition → Klotho activation → TGF-β1/Smad2/3 pathway suppression	db/db mice; HG-induced SV40-MES-13 cells	Reduced renal fibrosis markers, improved renal function, mitigated renal pathological changes	Zhang et al. (2024b)
		Notoginsenoside R1 (NGR1)	Activation of Nrf2/HO-1 pathway → inhibition of TGF-β1 signaling	db/db mice; HK-2 cells exposed to AGEs	Reduced ROS accumulation, apoptosis, and renal fibrosis; improved kidney histology	Zhang et al. (2019)
		LS-102 (Astragaloside IV derivative)	Inhibition of TGF-β1/Smad pathway; reduced M1 macrophage infiltration; downregulation of inflammatory factors	HFD-induced ORG rats; adipose stem cells *in vitro*	Reduced renal fibrosis, improved kidney function, decreased inflammation; superior effect compared to Astragaloside IV	Li et al. (2022)
Integrated Multi-Target Mechanisms	Flavonoids	Quercetin	PPARα activation; lipid metabolism; gut microbiota; gut–kidney axis	db/db mice; Uox^−/−^ rats	Enhances fatty acid oxidation; restores lipid homeostasis; modulates gut microbiota; reduces gut-derived uremic toxins; improves gut–kidney axis homeostasis	Guo et al. (2024); Peng et al. (2025)
		Apigenin	Uric acid metabolism; MAPK-mediated inflammatory signaling; CD38/SIRT3; TGF-β1/EMT axis; Wnt/β-catenin signaling	High uric acid (HUA) mice; HK-2 cells; STZ-induced DKD rats	Reduces serum uric acid; suppresses inflammation; alleviates oxidative stress; improves mitochondrial function; attenuates renal fibrosis	Liu et al. (2022b); Malik et al. (2017); Ogura et al. (2020); Li et al. (2021)
	Polysaccharides	Astragalus polysaccharide (APS)	Nrf2; TLR4/NLRP3/NF-κB; TGF-β/Smad; gut microbiota homeostasis	STZ-induced DKD rats; high glucose-treated podocytes	Modulates oxidative stress, inflammation, fibrosis, and gut microbiota homeostasis; improves renal injury	Guo et al. (2023)
	Alkaloids	Berberine (BBR)	NLRP3 inflammasome; EMT	DKD rats (HFD + STZ), HG-treated HK-2 cells	Suppresses inflammation and EMT; attenuates renal fibrotic remodeling; delays DKD progression	Ma et al. (2022)

## 4 Advancing natural product research through modern technology

Natural products have been used for the treatment of various diseases for centuries and remain a cornerstone of medical practice. Recent advancements in Chinese herbal medicine research in 2024 (Yan et al., 2025) demonstrate that natural products are highly effective in treating metabolic diseases. As modern technological methods continue to develop and evolve, the application of natural products has expanded, offering new approaches and perspectives for uncovering their mechanisms in the treatment of various systemic diseases. The convergence of multi-omics technologies, including network pharmacology, genomics, bioinformatics, and metabolomics (Zhu et al., 2022; Li et al., 2023), provides powerful tools for studying the multi-component, multi-target, and synergistic effects of natural products. Network pharmacology, which integrates drugs, targets, and disease systems into a “drug–target–disease” network, presents new opportunities for identifying the active ingredients and mechanisms through which natural products intervene in MDAKD. Thanks to its advantages of multi-component, multi-target actions and low toxicity, network pharmacology has become a widely used method in the study of natural products, offering theoretical support for the development of multi-target drugs and broadening the therapeutic strategies and discovery of potential indications for natural products (Li et al., 2017). Genomics involves analyzing the genetic makeup of organisms that produce natural products, identifying genes associated with secondary metabolites, and revealing the biosynthetic capabilities of these organisms. In contrast, metabolomics uses chemical profiling to identify the secondary metabolites actually produced, reflecting the overall phenotypic characteristics of the organism (Albarano et al., 2020). Moreover, recent advances in molecular biology have greatly accelerated the genomic sequencing of natural product-producing organisms, particularly bacteria and fungi (Caesar et al., 2021). Leveraging these technologies, computational approaches are increasingly used to predict interactions between compounds and targets (Wang et al., 2020), including molecular docking, molecular dynamics simulations, and other computer-aided drug design (CADD) techniques, which significantly enhance the efficiency and precision of active ingredient screening and mechanism analysis.

The increasing demand for natural product-based therapies, however, is hindered by the low concentrations of active ingredients and the challenges involved in their extraction and separation. As such, there is a pressing need for efficient and selective methods to address these barriers in drug development (Zhang et al., 2018). Building upon traditional techniques such as liquid-liquid extraction, solid-phase extraction, and solid-phase microextraction, numerous novel extraction technologies have emerged in recent years to more efficiently isolate bioactive compounds from plant sources. These include ultrasonic-assisted extraction, pressurized liquid extraction, subcritical water extraction, supercritical fluid extraction, microwave-assisted extraction, and instant controlled pressure-drop extraction (Yahya et al., 2018). These modern technologies not only enhance extraction efficiency and reduce energy consumption but also contribute to expanding our chemical understanding of natural products, thereby uncovering new sources of active compounds (Popova and Bankova, 2023). Looking ahead, artificial intelligence (AI) technologies, particularly machine learning and deep learning, are poised to revolutionize drug discovery from natural products (Saldívar-González et al., 2022). With the continuous expansion of natural product databases, AI-driven technologies have significantly improved the efficiency and accuracy of lead compound screening, accelerating the discovery of new drugs from natural products (Gangwal and Lavecchia, 2025). Therefore, the ongoing integration of AI is expected to unlock the therapeutic potential of natural products and drive the development of innovative therapies for MDAKD.

## 5 Conclusion and future directions

The paradigm of drug discovery in the 21st century has gradually shifted from single-target interventions toward the regulation of complex disease networks (Thomford et al., 2018). Owing to their remarkable chemical diversity and broad biological activities, natural products have become an important source of novel therapeutic agents. As a complex systemic disorder driven by metabolic dysfunction, MDAKD involves multiple interconnected pathogenic processes, including metabolic abnormalities, oxidative stress, chronic inflammation, gut microbiota dysbiosis, and renal fibrosis. This review systematically summarizes the major pathogenic mechanisms underlying MDAKD and recent advances in natural product-based interventions. Current evidence indicates that natural products exert renoprotective effects through multi-target, multi-pathway, and multi-level regulation of disease-associated networks. By restoring metabolic homeostasis, alleviating inflammation and oxidative stress, maintaining gut microbial balance, and preserving renal structure and function, natural products have demonstrated considerable therapeutic potential in MDAKD. Among them, polysaccharides, flavonoids, saponins, triterpenoids, and phenolic acids have all shown varying degrees of renoprotective activity, collectively highlighting the unique advantages of natural products in the multidimensional management of complex diseases.

Despite extensive preclinical evidence supporting the beneficial effects of natural products on MDAKD, their clinical translation remains in its infancy. Current clinical studies have primarily focused on DKD and diabetic albuminuria. Available evidence suggests that resveratrol, when used as an adjunct to RAAS blockade, can reduce urinary albumin excretion in patients with diabetic nephropathy (Sattarinezhad et al., 2019), whereas curcumin has been reported to improve proteinuria in patients with overt diabetic nephropathy (Vanaie et al., 2019). Although the current evidence remains limited, these findings provide preliminary clinical support for the potential role of natural products as adjunctive interventions in MDAKD management. These findings provide preliminary proof-of-concept evidence supporting the clinical potential of natural products in improving proteinuria, attenuating early renal injury, and optimizing metabolic status. Nevertheless, most available studies continue to rely on surrogate endpoints, such as UACR, proteinuria, inflammatory markers, and oxidative stress indicators, whereas evidence regarding hard clinical outcomes, including long-term eGFR decline, ESKD, cardiorenal composite outcomes, and mortality, remains limited. Furthermore, current clinical evidence is largely confined to DKD, while studies involving ORG and HN remain scarce. Therefore, natural products are presently better regarded as adjunctive interventions within the comprehensive management of MDAKD, and their long-term clinical benefits require further validation.

Safety assessment is equally important for the clinical development of natural products. Because many natural products are derived from medicinal foods, traditional herbal medicines, or naturally occurring dietary constituents and have a long history of human exposure, they are generally considered to possess favorable tolerability profiles. However, for chronic progressive diseases such as MDAKD, a natural origin does not necessarily guarantee long-term safety. Patients frequently receive concomitant therapies, including RAAS inhibitors, SGLT2 inhibitors, GLP-1RAs, as well as glucose-, lipid-, and uric acid-lowering agents. Consequently, the safety of natural products under conditions of prolonged administration, high-dose exposure, and combination therapy warrants careful evaluation. Existing studies have reported NOAELs of 200 mg/kg/day and 600 mg/kg/day for resveratrol in rats and dogs, respectively, providing preliminary evidence regarding its safety margin (Johnson et al., 2011). Overall, however, systematic toxicological investigations of natural products remain insufficient. Future studies should establish more comprehensive safety evaluation frameworks incorporating acute, subchronic, and chronic toxicity assessments, pharmacokinetic analyses, histopathological examinations, and long-term tolerability studies, thereby providing a more robust basis for balancing efficacy and safety (Xu et al., 2020).

Another issue that deserves particular attention is the limited characterization of dose–response relationships. Although many studies have demonstrated therapeutic efficacy, systematic investigations examining dose–response patterns remain relatively scarce. While dose-dependent renoprotective effects have been reported for certain natural products, substantial variations in dosage ranges, intervention durations, and outcome measures across studies have hindered the establishment of consensus regarding optimal therapeutic doses, minimum effective doses, and intervention windows. Furthermore, whether prolonged administration at higher doses can further enhance efficacy or increase the risk of adverse effects remains unclear. Future studies should therefore incorporate standardized dose-gradient designs, pharmacodynamic evaluations, and long-term follow-up analyses to establish more reliable dosing strategies and facilitate subsequent clinical translation.

Despite their considerable therapeutic potential in MDAKD, several critical challenges continue to hinder the further development and application of natural products. Owing to their complex chemical composition, variations in extraction and purification procedures may substantially alter the content and composition of bioactive constituents, thereby affecting the stability, reproducibility, and comparability of research findings (Zhang et al., 2026). In addition, residual impurities and interference from non-target compounds may further complicate quality control and pharmacological evaluation. Many natural products also exhibit unfavorable pharmacokinetic characteristics, including poor oral absorption, extensive first-pass metabolism, limited aqueous or lipid solubility, and low bioavailability, all of which may restrict effective systemic exposure and sustained therapeutic efficacy (Hu, 2023). Furthermore, considerable heterogeneity exists among studies regarding formulation types, routes of administration, and dosing regimens, with no universally accepted standards currently available, thereby complicating cross-study comparisons and evidence integration (Chen et al., 2018). Therefore, future efforts should focus on optimizing extraction and purification procedures, strengthening quality-control systems, improving pharmacokinetic evaluation, and standardizing dosing strategies to enhance the reproducibility and comparability of research findings and facilitate the standardized evaluation and clinical application of natural products. Furthermore, natural product research is gradually transitioning from an experience-based paradigm toward evidence-based (Li et al., 2026), standardized, and precision-oriented development, which may provide a stronger theoretical and technological foundation for mechanistic elucidation, quality control, and clinical translation in MDAKD. Meanwhile, the rapid advancement of multi-omics technologies, systems biology, network pharmacology, and artificial intelligence-assisted drug discovery is creating new opportunities for natural product research (Wu et al., 2026). By integrating multidimensional biological datasets and constructing component–target–disease interaction networks, these approaches may enable a more systematic elucidation of the mechanisms underlying natural product interventions, facilitate the identification of key bioactive constituents, and improve the accuracy of target prediction (Liu and Zeng, 2025). Collectively, these advances are expected to enhance the reliability and translational efficiency of natural product research, thereby providing important technological support for the standardized development of natural products and the establishment of precision intervention strategies for MDAKD.

Particularly noteworthy is the potential synergy between natural products and currently available renoprotective therapies, which may provide new opportunities for the integrated management of MDAKD. In recent years, therapeutic strategies for complex diseases have increasingly shifted from single-agent interventions toward precision-oriented and combination-based approaches aimed at achieving greater long-term clinical benefits (Hussain et al., 2025), a trend that may also provide new perspectives for integrating natural products into comprehensive MDAKD management. Existing evidence suggests that natural products may be more suitable as adjunctive therapies rather than substitutes for established evidence-based treatments. For instance, supplementation with green tea polyphenols has been shown to further reduce albuminuria in patients with DKD who continue to exhibit residual albuminuria despite treatment with ACEIs or ARBs, suggesting potential benefits of combination therapy (Borges et al., 2016). As SGLT2 inhibitors and GLP-1RAs become increasingly important components of CKD management in patients with diabetes, future investigations should extend beyond traditional RAAS blockade and explore combination strategies involving these emerging renoprotective agents. Mechanistically, SGLT2 inhibitors primarily improve glomerular hyperfiltration, tubular energy burden, and hemodynamic abnormalities, whereas GLP-1RAs exert beneficial effects on glycemic control, body weight reduction, and cardiorenal metabolic risk (Rossing et al., 2022). In contrast, natural products target multiple residual injury pathways through antioxidative, anti-inflammatory, microbiota-regulating, mitochondria-protective, and antifibrotic actions. Such mechanistic complementarity raises the possibility of additive or even synergistic therapeutic benefits. Future randomized controlled trials should therefore evaluate whether adjunctive natural product interventions can further improve UACR, eGFR decline, renal fibrosis markers, and cardiorenal composite outcomes while simultaneously assessing drug interactions and long-term tolerability.

Overall, research on natural products is reshaping our understanding of therapeutic strategies for MDAKD. Unlike conventional interventions that target a single molecule or signaling pathway, natural products exert broader regulatory effects across interconnected pathogenic networks involving metabolic dysfunction, inflammation, oxidative stress, gut microbiota dysbiosis, and fibrosis. This network-based mode of action is highly consistent with the multifactorial nature of MDAKD. Current evidence suggests that natural products not only ameliorate multiple pathogenic processes but may also provide more comprehensive renoprotection through multidimensional synergistic regulation. As mechanistic insights continue to deepen and the quality of both preclinical and clinical evidence improves, natural products are expected to become an increasingly important component of integrated MDAKD management and may offer new opportunities for the precision intervention of metabolic kidney diseases.

## References

[B1] Abasheva D. Ortiz A. Fernandez-Fernandez B. (2024). GLP-1 receptor agonists in patients with chronic kidney disease and either overweight or obesity. Clin. Kidney J. 17, ii19–ii35. 10.1093/ckj/sfae296 PMC11581768 39583142

[B2] Ajila C. M. Brar S. K. Verma M. Tyagi R. D. Godbout S. Valéro J. R. (2011). Extraction and analysis of polyphenols: recent trends. Crit. Rev. Biotechnol. 31, 227–249. 10.3109/07388551.2010.513677 21073258

[B3] Akram M. (2014). Citric acid cycle and role of its intermediates in metabolism. Cell Biochem. Biophys. 68, 475–478. 10.1007/s12013-013-9750-1 24068518

[B4] Albarano L. Esposito R. Ruocco N. Costantini M. (2020). Genome mining as new challenge in natural products discovery. Mar. Drugs 18, 199. 10.3390/md18040199 32283638 PMC7230286

[B5] Anders H.-J. Andersen K. Stecher B. (2013). The intestinal microbiota, a leaky gut, and abnormal immunity in kidney disease. Kidney Int. 83, 1010–1016. 10.1038/ki.2012.440 23325079

[B6] Baldwin W. McRae S. Marek G. Wymer D. Pannu V. Baylis C. (2011). Hyperuricemia as a mediator of the proinflammatory endocrine imbalance in the adipose tissue in a murine model of the metabolic syndrome. Diabetes 60, 1258–1269. 10.2337/db10-0916 21346177 PMC3064099

[B7] Bansal A. Chonchol M. (2025). Metabolic dysfunction-associated kidney disease: pathogenesis and clinical manifestations. Kidney International 108, 194–200. 10.1016/j.kint.2025.01.044 40379048

[B8] Baptista F. Paié-Ribeiro J. Almeida M. Barros A. N. (2024). Exploring the role of phenolic compounds in chronic kidney disease: a systematic review. Molecules 29, 2576. 10.3390/molecules29112576 38893451 PMC11173950

[B9] Bhargava P. Schnellmann R. G. (2017). Mitochondrial energetics in the kidney. Nat. Rev. Nephrol. 13, 629–646. 10.1038/nrneph.2017.107 28804120 PMC5965678

[B10] Bilan M. I. Usov A. I. (2008). Structural analysis of fucoidans. Nat. Product. Commun. 3, 1934578X0800301011. 10.1177/1934578X0800301011

[B11] Boeing T. Reis Lívero F. A. D. de Souza P. de Almeida D. A. T. Donadel G. Lourenço E. L. B. (2023). Natural products as modulators of mitochondrial dysfunctions associated with cardiovascular diseases: advances and opportunities. J. Med. Food 26, 279–298. 10.1089/jmf.2022.0022 37186894

[B12] Borges C. M. Papadimitriou A. Duarte D. A. Lopes De Faria J. M. Lopes De Faria J. B. (2016). The use of green tea polyphenols for treating residual albuminuria in diabetic nephropathy: a double-blind randomised clinical trial. Sci. Rep. 6, 28282. 10.1038/srep28282 27320846 PMC4913255

[B13] Caesar L. K. Montaser R. Keller N. P. Kelleher N. L. (2021). Metabolomics and genomics in natural products research: complementary tools for targeting new chemical entities. Nat. Prod. Rep. 38, 2041–2065. 10.1039/d1np00036e 34787623 PMC8691422

[B14] Cao C. Zhu H. Yao Y. Zeng R. (2022). Gut dysbiosis and kidney diseases. Front. Med. (Lausanne) 9, 829349. 10.3389/fmed.2022.829349 35308555 PMC8927813

[B15] Chang J. Yan J. Li X. Liu N. Zheng R. Zhong Y. (2021). Update on the mechanisms of tubular cell injury in diabetic kidney disease. Front. Med. (Lausanne) 8, 661076. 10.3389/fmed.2021.661076 33859992 PMC8042139

[B16] Chang Z. Xu X. Xu Y. Wei X. Yang G. Guo P. (2025). Potential effects of functional foods and dietary supplements on metabolic-associated fatty liver disease and the underlying mechanisms: a narrative review with a focus on the modulation of the gut microbiota. J. Agric. Food Chem. 73, 29247–29280. 10.1021/acs.jafc.5c05587 41212763

[B17] Chaudhuri J. Bains Y. Guha S. Kahn A. Hall D. Bose N. (2018). The role of advanced glycation end products in aging and metabolic diseases: bridging association and causality. Cell Metab. 28, 337–352. 10.1016/j.cmet.2018.08.014 30184484 PMC6355252

[B18] Chen D.-Q. Hu H.-H. Wang Y.-N. Feng Y.-L. Cao G. Zhao Y.-Y. (2018). Natural products for the prevention and treatment of kidney disease. Phytomedicine 50, 50–60. 10.1016/j.phymed.2018.09.182 30466992

[B19] Chen J. Tsim K. W. K. Gao K. Khazaeli M. (2022). Editorial: the gut-kidney axis: a potential drug target for treating kidney diseases. Front. Pharmacol. 13, 1012890. 10.3389/fphar.2022.1012890 36160456 PMC9490406

[B20] Chen Z. Liang H. Yan X. Liang Q. Bai Z. Xie T. (2023). Astragalus polysaccharide promotes autophagy and alleviates diabetic nephropathy by targeting the lncRNA Gm41268/PRLR pathway. Ren. Fail. 45, 2284211. 10.1080/0886022X.2023.2284211 37994436 PMC11001349

[B21] Chen Y. Li H. Lai F. Min T. Wu H. Zhan Q. (2024). The influence and mechanisms of natural plant polysaccharides on intestinal microbiota-mediated metabolic disorders. Foods 13, 3882. 10.3390/foods13233882 39682954 PMC11640612

[B22] Chenxu G. Xianling D. Qin K. Linfeng H. Yan S. Mingxin X. (2021). Fisetin protects against high fat diet-induced nephropathy by inhibiting inflammation and oxidative stress *via* the blockage of iRhom2/NF-κB signaling. Int. Immunopharmacol. 92, 107353. 10.1016/j.intimp.2020.107353 33429334

[B23] Chrysopoulou M. Rinschen M. M. (2024). Metabolic rewiring and communication: an integrative view of kidney proximal tubule function. Annu. Rev. Physiol. 86, 405–427. 10.1146/annurev-physiol-042222-024724 38012048

[B24] Dai H. Shan Z. Shi L. Duan Y. An Y. He C. (2024). Mulberry leaf polysaccharides ameliorate glucose and lipid metabolism disorders *via* the gut microbiota-bile acids metabolic pathway. Int. J. Biol. Macromol. 282, 136876. 10.1016/j.ijbiomac.2024.136876 39490871

[B25] de Vries A. P. J. Ruggenenti P. Ruan X. Z. Praga M. Cruzado J. M. Bajema I. M. (2014). Fatty kidney: emerging role of ectopic lipid in obesity-related renal disease. Lancet Diabetes Endocrinol. 2, 417–426. 10.1016/S2213-8587(14)70065-8 24795255

[B26] Delgado-Andrade C. (2016). Carboxymethyl-lysine: thirty years of investigation in the field of AGE formation. Food Funct. 7, 46–57. 10.1039/c5fo00918a 26462729

[B27] Diaconeasa Z. Leopold L. Rugină D. Ayvaz H. Socaciu C. (2015). Antiproliferative and antioxidant properties of anthocyanin rich extracts from blueberry and blackcurrant juice. Int. J. Mol. Sci. 16, 2352–2365. 10.3390/ijms16022352 25622252 PMC4346840

[B28] Dong H. Qi X. (2025). Biosynthesis of triterpenoids in plants: pathways, regulation, and biological functions. Curr. Opin. Plant Biol. 85, 102701. 10.1016/j.pbi.2025.102701 40112428

[B29] Dong W. Zhao Y. Li X. Huo J. Wang W. (2023). Corn silk polysaccharides attenuate diabetic nephropathy through restoration of the gut microbial ecosystem and metabolic homeostasis. Front. Endocrinol. 14, 1232132. 10.3389/fendo.2023.1232132 PMC10726137 38111708

[B30] Dozio E. Caldiroli L. Molinari P. Castellano G. Delfrate N. W. Romanelli M. M. C. (2023). Accelerated AGEing: the impact of advanced glycation end products on the prognosis of chronic kidney disease. Antioxidants (Basel) 12, 584. 10.3390/antiox12030584 36978832 PMC10045600

[B31] Du L. Zong Y. Li H. Wang Q. Xie L. Yang B. (2024). Hyperuricemia and its related diseases: mechanisms and advances in therapy. Signal Transduct. Target. Ther. 9, 212. 10.1038/s41392-024-01916-y 39191722 PMC11350024

[B32] Ediga H. H. Hester P. Yepuri A. Reddy G. B. Madala S. K. (2023). Nε-Carboxymethyl-Lysine modification of extracellular matrix proteins augments fibroblast activation. Int. J. Mol. Sci. 24, 15811. 10.3390/ijms242115811 37958795 PMC10650592

[B33] Evenepoel P. Poesen R. Meijers B. (2017). The gut-kidney axis. Pediatr. Nephrol. 32, 2005–2014. 10.1007/s00467-016-3527-x 27848096

[B34] Fan Y. Pedersen O. (2021). Gut microbiota in human metabolic health and disease. Nat. Rev. Microbiol. 19, 55–71. 10.1038/s41579-020-0433-9 32887946

[B35] Fan Z. Wei X. Zhu X. Yang K. Tian L. Wang X. (2025). Unveiling the therapeutic potential of berberine: its therapeutic role and molecular mechanisms in kidney diseases. Front. Pharmacol. 16, 1549462. 10.3389/fphar.2025.1549462 40061955 PMC11885249

[B36] Feng L. Gu C. Li Y. Huang J. (2017). High glucose promotes CD36 expression by upregulating peroxisome proliferator-activated receptor *γ* levels to exacerbate lipid deposition in renal tubular cells. BioMed Res. Int. 2017, 1–10. 10.1155/2017/1414070 28497039 PMC5405368

[B37] Feng Y. Weng H. Ling L. Zeng T. Zhang Y. Chen D. (2019). Modulating the gut microbiota and inflammation is involved in the effect of bupleurum polysaccharides against diabetic nephropathy in mice. Int. J. Biol. Macromol. 132, 1001–1011. 10.1016/j.ijbiomac.2019.03.242 30946910

[B38] Ferrare K. Bidel L. P. R. Awwad A. Poucheret P. Cazals G. Lazennec F. (2018). Increase in insulin sensitivity by the association of chicoric acid and chlorogenic acid contained in a natural chicoric acid extract (NCRAE) of chicory (Cichorium intybus L.) for an antidiabetic effect. J. Ethnopharmacol. 215, 241–248. 10.1016/j.jep.2017.12.035 29325917

[B39] Figueras T. Perdicaro D. J. Cacciamani V. E. Gil Lorenzo A. F. Suhaiman L. Antoniolli A. N. (2025). Grape pomace extract, rich in phenolic compounds, attenuates obesity-induced nephropathy by modulating energy metabolism dysregulation and oxidative stress in mice fed a high-fat diet. Food Funct. 16, 6833–6847. 10.1039/D5FO02327K 40767161

[B40] Forbes J. M. Thorburn D. R. (2018). Mitochondrial dysfunction in diabetic kidney disease. Nat. Rev. Nephrol. 14, 291–312. 10.1038/nrneph.2018.9 29456246

[B41] Foresto-Neto O. Menezes-Silva L. Leite J. A. Andrade-Silva M. Câmara N. O. S. (2024). Immunology of kidney disease. Annu. Rev. Immunol. 42, 207–233. 10.1146/annurev-immunol-090122-045843 38211945

[B42] Forrester S. J. Kikuchi D. S. Hernandes M. S. Xu Q. Griendling K. K. (2018). Reactive oxygen species in metabolic and inflammatory signaling. Circ. Res. 122, 877–902. 10.1161/CIRCRESAHA.117.311401 29700084 PMC5926825

[B43] Fotheringham A. K. Gallo L. A. Borg D. J. Forbes J. M. (2022). Advanced glycation end products (AGEs) and chronic kidney disease: does the modern diet AGE the kidney? Nutrients 14, 2675. 10.3390/nu14132675 35807857 PMC9268915

[B44] Gai Z. Wang T. Visentin M. Kullak-Ublick G. A. Fu X. Wang Z. (2019). Lipid accumulation and chronic kidney disease. Nutrients 11, 722. 10.3390/nu11040722 30925738 PMC6520701

[B45] Gangwal A. Lavecchia A. (2025). Artificial intelligence in natural product drug discovery: current applications and future perspectives. J. Med. Chem. 68, 3948–3969. 10.1021/acs.jmedchem.4c01257 39916476 PMC11874025

[B46] Garud M. S. Kulkarni Y. A. (2018). Gallic acid attenuates type I diabetic nephropathy in rats. Chem. Biol. Interact. 282, 69–76. 10.1016/j.cbi.2018.01.010 29331653

[B47] Gerich J. E. (2010). Role of the kidney in normal glucose homeostasis and in the hyperglycaemia of diabetes mellitus: therapeutic implications. Diabet. Med. 27, 136–142. 10.1111/j.1464-5491.2009.02894.x 20546255 PMC4232006

[B48] Gewin L. S. (2018). Renal fibrosis: primacy of the proximal tubule. Matrix Biol. 68 (69), 248–262. 10.1016/j.matbio.2018.02.006 29425694 PMC6015527

[B49] Gherghina M.-E. Peride I. Tiglis M. Neagu T. P. Niculae A. Checherita I. A. (2022). Uric acid and oxidative stress—relationship with cardiovascular, metabolic, and renal impairment. IJMS 23, 3188. 10.3390/ijms23063188 35328614 PMC8949471

[B50] Guo C. Liang T. He Q. Wei P. Zheng N. Xu L. (2013). Renoprotective effect of ramulus mori polysaccharides on renal injury in STZ-diabetic mice. Int. J. Biol. Macromol. 62, 720–725. 10.1016/j.ijbiomac.2013.09.022 24076200

[B51] Guo M. Gao J. Jiang L. Dai Y. (2023). Astragalus polysaccharide ameliorates renal inflammatory responses in a diabetic nephropathy by suppressing the TLR4/NF-κB pathway. Drug Des. Devel Ther. 17, 2107–2118. 10.2147/DDDT.S411211 PMC10363349 37489175

[B52] Guo X. Wen S. Wang J. Zeng X. Yu H. Chen Y. (2024). Senolytic combination of dasatinib and quercetin attenuates renal damage in diabetic kidney disease. Phytomedicine 130, 155705. 10.1016/j.phymed.2024.155705 38761776

[B53] Guo J. Liu H. Jin T. Jia J. Zhu W. Xia X. (2025). Moslae herba extract alleviates hyperuricemia by regulating uric acid metabolism and relieving renal inflammation and fibrosis in mice. Phytomedicine 145, 156974. 10.1016/j.phymed.2025.156974 40541125

[B54] Han Q. Chen N. Peng K. Wang M. Chen M. Mamadalieva N. Z. (2025). Urolithin C ameliorates hyperuricemia by mitigating renal dysfunction through ROS-p38/ERK MAPK axis suppression. J. Agric. Food Chem. 73, 22485–22501. 10.1021/acs.jafc.5c09146 40890082

[B55] Ho Do M. Seo Y. S. Park H.-Y. (2021). Polysaccharides: bowel health and gut microbiota. Crit. Rev. Food Sci. Nutr. 61, 1212–1224. 10.1080/10408398.2020.1755949 32319786

[B56] Holmes Z. C. Silverman J. D. Dressman H. K. Wei Z. Dallow E. P. Armstrong S. C. (2020). Short-chain fatty acid production by gut microbiota from children with obesity differs according to prebiotic choice and bacterial community composition. mBio 11, e00914–e00920. 10.1128/mBio.00914-20 32788375 PMC7439474

[B57] Hosseini A. Razavi B. M. Banach M. Hosseinzadeh H. (2021). Quercetin and metabolic syndrome: a review. Phytother. Res. 35, 5352–5364. 10.1002/ptr.7144 34101925

[B58] Hou Y. Wang Q. Han B. Chen Y. Qiao X. Wang L. (2021). CD36 promotes NLRP3 inflammasome activation *via* the mtROS pathway in renal tubular epithelial cells of diabetic kidneys. Cell Death Dis. 12, 523. 10.1038/s41419-021-03813-6 34021126 PMC8140121

[B59] Hu Q. (2023). A Natural Products Solution to Diabetic Nephropathy Therapy. 10.1016/j.pharmthera.2022.108314 36427568

[B60] Hu Y. Wang S.-X. Wu F.-Y. Wu K.-J. Shi R.-P. Qin L.-H. (2022). Effects and mechanism of Ganoderma lucidum polysaccharides in the treatment of diabetic nephropathy in streptozotocin-induced diabetic rats. Biomed. Res. Int. 2022, 4314415. 10.1155/2022/4314415 35299891 PMC8923773

[B61] Hu R. Zhao Z. Xie L. Ma Z. Wu W. Li S. (2025). Global, regional, and national burden of chronic kidney disease due to diabetes mellitus type 2 from 1990 to 2021, with projections to 2036: a systematic analysis for the global Burden of Disease Study 2021. Front. Med. 12, 1531811. 10.3389/fmed.2025.1531811 PMC11872908 40034386

[B62] Hussain J. M. Khan S. M. Sultan H. Rathi D. Woldeyohannes E. M. Alemayehu Z. G. (2025). The role of immune checkpoint inhibitors in adrenocortical carcinoma: enhancing post‐surgical outcomes and overcoming relapse. Med. Adv. 3, 205–208. 10.1002/med4.70019

[B63] Im P. K. Kartsonaki C. Kakkoura M. G. Mohamed-Ahmed O. Yang L. Chen Y. (2025). Hyperuricemia, gout and the associated comorbidities in China: findings from a prospective study of 0.5 million adults. Lancet Regional Health - West. Pac. 58, 101572. 10.1016/j.lanwpc.2025.101572 PMC12167516 40519626

[B64] Iwano M. Plieth D. Danoff T. M. Xue C. Okada H. Neilson E. G. (2002). Evidence that fibroblasts derive from epithelium during tissue fibrosis. J. Clin. Invest 110, 341–350. 10.1172/JCI15518 12163453 PMC151091

[B65] Jiang Y.-Y. Jiang X.-L. Yu H.-N. (2025). Dysregulation of lipid metabolism in chronic kidney disease and the role of natural products. Schmiedeb. Arch. Pharmacol. 398, 261–278. 10.1007/s00210-024-03373-4 39162795

[B66] Jiao J. Yong T. Huang L. Chen S. Xiao C. Wu Q. (2023). A Ganoderma lucidum polysaccharide F31 alleviates hyperglycemia through kidney protection and adipocyte apoptosis. Int. J. Biol. Macromol. 226, 1178–1191. 10.1016/j.ijbiomac.2022.11.231 36442553

[B67] Johnson W. D. Morrissey R. L. Usborne A. L. Kapetanovic I. Crowell J. A. Muzzio M. (2011). Subchronic oral toxicity and cardiovascular safety pharmacology studies of resveratrol, a naturally occurring polyphenol with cancer preventive activity. Food Chem. Toxicol. 49, 3319–3327. 10.1016/j.fct.2011.08.023 21939727 PMC3223276

[B68] Kang H. M. Ahn S. H. Choi P. Ko Y. A. Han S. H. Chinga F. (2015). Defective fatty acid oxidation in renal tubular epithelial cells has a key role in kidney fibrosis development. Nat. Med. 21, 37–46. 10.1038/nm.3762 25419705 PMC4444078

[B69] Kimura Y. Tsukui D. Kono H. (2021). Uric acid in inflammation and the pathogenesis of atherosclerosis. IJMS 22, 12394. 10.3390/ijms222212394 34830282 PMC8624633

[B70] Koh E. S. Lim J. H. Kim M. Y. Chung S. Shin S. J. Choi B. S. (2015). Anthocyanin-rich seoritae extract ameliorates renal lipotoxicity *via* activation of AMP-Activated protein kinase in diabetic mice. J. Transl. Med. 13, 203. 10.1186/s12967-015-0563-4 26116070 PMC4482313

[B71] Koska J. Gerstein H. C. Beisswenger P. J. Reaven P. D. (2022). Response to comment on koska et al. Advanced glycation end products predict loss of renal function and high-risk chronic kidney disease in type 2 diabetes. Diabetes care 2022;44:684-691. Diabetes Care 45, e111–e112. 10.2337/dci22-0013 35653599 PMC9210513

[B72] Kurts C. Panzer U. Anders H.-J. Rees A. J. (2013). The immune system and kidney disease: basic concepts and clinical implications. Nat. Rev. Immunol. 13, 738–753. 10.1038/nri3523 24037418

[B73] Laorodphun P. Arjinajarn P. Thongnak L. Promsan S. Swe M. T. Thitisut P. (2021). Anthocyanin‐rich fraction from black rice, *Oryza sativa* L. var. indica “Luem Pua,” bran extract attenuates kidney injury induced by high‐fat diet involving oxidative stress and apoptosis in obese rats. Phytother. Res. 35, 5189–5202. 10.1002/ptr.7188 34327741

[B74] Lee A.-T. Yang M.-Y. Tsai I.-N. Chang Y.-C. Hung T.-W. Wang C.-J. (2024a). Gallic acid alleviates glucolipotoxicity-induced nephropathy by miR-709-NFE2L2 pathway in db/db mice on a high-fat diet. J. Agric. Food Chem. 72, 22645–22660. 10.1021/acs.jafc.4c05898 39365293 PMC11487656

[B75] Lee L. E. Doke T. Mukhi D. Susztak K. (2024b). The key role of altered tubule cell lipid metabolism in kidney disease development. Kidney Int. 106, 24–34. 10.1016/j.kint.2024.02.025 38614389 PMC11193624

[B76] Levassort H. Essig M. (2024). The kidney, its anatomy and main functions. Soins Gerontol. 29, 10–20. 10.1016/j.sger.2023.12.003 38331520

[B77] Li W. Yuan G. Pan Y. Wang C. Chen H. (2017). Network pharmacology studies on the bioactive compounds and action mechanisms of natural products for the treatment of diabetes mellitus: a review. Front. Pharmacol. 08, 74. 10.3389/fphar.2017.00074 28280467 PMC5322182

[B78] Li Y. Zhao Z. Luo J. Jiang Y. Li L. Chen Y. (2021). Apigenin ameliorates hyperuricemic nephropathy by inhibiting URAT1 and GLUT9 and relieving renal fibrosis *via* the wnt/β-catenin pathway. Phytomedicine 87, 153585. 10.1016/j.phymed.2021.153585 34044255

[B79] Li Z. Yang W. Yang Y. Wu J. Luo P. Liu Y. (2022). The astragaloside IV derivative LS-102 ameliorates obesity-related nephropathy. Drug Des. Devel Ther. 16, 647–664. 10.2147/DDDT.S346546 PMC8932932 35308255

[B80] Li J.-Z. Chen N. Ma N. Li M.-R. (2023). Mechanism and progress of natural products in the treatment of NAFLD-related fibrosis. Molecules 28, 7936. 10.3390/molecules28237936 38067665 PMC10707854

[B81] Li M.-Y. Wu G.-F. Lu F. Geng C. Yu S. Gao M.-L. (2026). Food and Medicine homology focus in 2026. Food & Med. Homol. 3, 9420133. 10.26599/FMH.2026.9420133

[B82] Lian Y. Zhu M. Chen J. Yang B. Lv Q. Wang L. (2021). Characterization of a novel polysaccharide from moutan cortex and its ameliorative effect on AGEs-induced diabetic nephropathy. Int. J. Biol. Macromol. 176, 589–600. 10.1016/j.ijbiomac.2021.02.062 33581205

[B83] Liang S. Xu D. Wu J. Jiang Q. Zeng Y. (2024). Phyllanthi fructus ameliorates hyperuricemia and kidney injure *via* inhibiting uric acid synthesis, modulating urate transporters, and alleviating inflammation. Sci. Rep. 14, 27605. 10.1038/s41598-024-79350-x 39528682 PMC11555318

[B84] Lin M. Yiu W. H. Wu H. J. Chan L. Y. Y. Leung J. C. K. Au W. S. (2012). Toll-like receptor 4 promotes tubular inflammation in diabetic nephropathy. J. Am. Soc. Nephrol. 23, 86–102. 10.1681/ASN.2010111210 22021706 PMC3269929

[B85] Lin H.-H. Liang Y.-H. Chyau C.-C. Tseng C.-Y. Zhang J.-Q. Chen J.-H. (2025). Desmodium caudatum (thunb.) DC. extract attenuates hyperuricemia-induced renal fibrosis *via* modulating TGF-β1 pathway and uric acid transporters: evidence from *in vitro* and *in vivo* studies. J. Ethnopharmacol. 345, 119609. 10.1016/j.jep.2025.119609 40064319

[B86] Listenberger L. L. Han X. Lewis S. E. Cases S. Farese R. V. Ory D. S. (2003). Triglyceride accumulation protects against fatty acid-induced lipotoxicity. Proc. Natl. Acad. Sci. U. S. A. 100, 3077–3082. 10.1073/pnas.0630588100 12629214 PMC152249

[B87] Liu Y. (2011). Cellular and molecular mechanisms of renal fibrosis. Nat. Rev. Nephrol. 7, 684–696. 10.1038/nrneph.2011.149 22009250 PMC4520424

[B88] Liu T.-T. Zeng K.-W. (2025). Recent advances in target identification technology of natural products. Pharmacol. Ther. 269, 108833. 10.1016/j.pharmthera.2025.108833 40015520

[B89] Liu X. Wang W. Song G. Wei X. Zeng Y. Han P. (2017). Astragaloside IV ameliorates diabetic nephropathy by modulating the mitochondrial quality control network. PLoS One 12, e0182558. 10.1371/journal.pone.0182558 28767702 PMC5540580

[B90] Liu L. Ning X. Wei L. Zhou Y. Zhao L. Ma F. (2022a). Twist1 downregulation of PGC-1α decreases fatty acid oxidation in tubular epithelial cells, leading to kidney fibrosis. Theranostics 12, 3758–3775. 10.7150/thno.71722 35664054 PMC9131259

[B91] Liu T. Gao H. Zhang Y. Wang S. Lu M. Dai X. (2022b). Apigenin ameliorates hyperuricemia and renal injury through regulation of uric acid metabolism and JAK2/STAT3 signaling pathway. Pharm. (Basel) 15, 1442. 10.3390/ph15111442 PMC9697024 36422572

[B92] Liu Y. Hu Z. Xing H. Kang L. Chen X. Liu B. (2022c). Renoprotective effects of oleanolic acid and its possible mechanisms in rats with diabetic kidney disease. Biochem. Biophysical Res. Commun. 636, 1–9. 10.1016/j.bbrc.2022.10.074 36332469

[B93] Liu Y. Zheng K. Wang H. Liu H. Zheng K. Zhang J. (2024). Natural bioactive compounds: emerging therapies for hyperuricemia. Am. J. Chin. Med. 52, 1863–1885. 10.1142/S0192415X24500733 39558557

[B94] Lu H.-J. Tzeng T.-F. Liou S.-S. Da Lin S. Wu M.-C. Liu I.-M. (2014). Polysaccharides from liriopes radix ameliorate streptozotocin-induced type I diabetic nephropathy *via* regulating NF-κB and p38 MAPK signaling pathways. BMC Complement. Altern. Med. 14, 156. 10.1186/1472-6882-14-156 24886259 PMC4041058

[B95] Lu Y.-P. Wang X.-H. Xia B. Wu H.-W. Lei Y. Cai K.-W. (2025). C3G improves lipid droplet accumulation in the proximal tubules of high-fat diet-induced ORG mice. Pharmacol. Res. 211, 107550. 10.1016/j.phrs.2024.107550 39675540

[B96] Lymperopoulos A. Suster M. S. Borges J. I. (2022). Short-chain fatty acid receptors and cardiovascular function. Int. J. Mol. Sci. 23, 3303. 10.3390/ijms23063303 35328722 PMC8952772

[B97] Lytrivi M. Castell A.-L. Poitout V. Cnop M. (2020). Recent insights into mechanisms of β-Cell Lipo- and glucolipotoxicity in type 2 diabetes. J. Mol. Biol. 432, 1514–1534. 10.1016/j.jmb.2019.09.016 31628942 PMC7073302

[B98] Ma Z. Zhu L. Wang S. Guo X. Sun B. Wang Q. (2022). Berberine protects diabetic nephropathy by suppressing epithelial-to-mesenchymal transition involving the inactivation of the NLRP3 inflammasome. Ren. Fail. 44, 923–932. 10.1080/0886022x.2022.2079525 35618411 PMC9154812

[B99] Ma N. Cai S. Sun Y. Chu C. (2024). Chinese sumac (rhus chinensis mill.) fruits prevent hyperuricemia and uric acid nephropathy in mice fed a high-purine yeast diet. Nutrients 16, 184. 10.3390/nu16020184 38257077 PMC10819650

[B100] Ma Y. Wang X. Lin S. King L. Liu L. (2025). The potential role of advanced glycation end products in the development of kidney disease. Nutrients 17, 758. 10.3390/nu17050758 40077627 PMC11902189

[B101] Malik S. Suchal K. Khan S. I. Bhatia J. Kishore K. Dinda A. K. (2017). Apigenin ameliorates streptozotocin-induced diabetic nephropathy in rats *via* MAPK-NF-κB-TNF-α and TGF-β1-MAPK-fibronectin pathways. Am. J. Physiol. Ren. Physiol. 313, F414–F422. 10.1152/ajprenal.00393.2016 28566504

[B102] Martínez Montoro J. I. Morales E. Cornejo Pareja I. Tinahones F. J. Fernández García J. C. (2022). Obesity related glomerulopathy: current approaches and future perspectives. Obes. Rev. 23, e13450. 10.1111/obr.13450 35362662 PMC9286698

[B103] Martins M. Ribeiro M. H. Almeida C. M. M. (2023). Physicochemical, nutritional, and medicinal properties of Opuntia ficus-indica (L.) mill. and its main agro-industrial use: a review. Plants (Basel) 12, 1512. 10.3390/plants12071512 37050137 PMC10096643

[B104] Mei Y. Dong B. Geng Z. Xu L. (2022). Excess uric acid induces gouty nephropathy through crystal formation: a review of recent insights. Front. Endocrinol. (Lausanne) 13, 911968. 10.3389/fendo.2022.911968 35909538 PMC9329685

[B105] Miguel V. Shaw I. W. Kramann R. (2025). Metabolism at the crossroads of inflammation and fibrosis in chronic kidney disease. Nat. Rev. Nephrol. 21, 39–56. 10.1038/s41581-024-00889-z 39289568

[B106] Minami S. Sakai S. Yamamoto T. Takabatake Y. Namba-Hamano T. Takahashi A. (2024). FGF21 and autophagy coordinately counteract kidney disease progression during aging and obesity. Autophagy 20, 489–504. 10.1080/15548627.2023.2259282 37722816 PMC10936614

[B107] Mitrofanova A. Merscher S. Fornoni A. (2023). Kidney lipid dysmetabolism and lipid droplet accumulation in chronic kidney disease. Nat. Rev. Nephrol. 19, 629–645. 10.1038/s41581-023-00741-w 37500941 PMC12926870

[B108] Mojadami S. Ahangarpour A. Mard S. A. Khorsandi L. (2023). Diabetic nephropathy induced by methylglyoxal: gallic acid regulates kidney microRNAs and glyoxalase1–Nrf2 in male mice. Archives Physiology Biochem. 129, 655–662. 10.1080/13813455.2020.1857775 33460343

[B109] Moudgil K. D. Venkatesha S. H. (2022). The anti-inflammatory and immunomodulatory activities of natural products to control autoimmune inflammation. Int. J. Mol. Sci. 24, 95. 10.3390/ijms24010095 36613560 PMC9820125

[B110] Nakamura J. Yamamoto T. Takabatake Y. Namba-Hamano T. Minami S. Takahashi A. (2023). TFEB-mediated lysosomal exocytosis alleviates high-fat diet-induced lipotoxicity in the kidney. JCI Insight 8, e162498. 10.1172/jci.insight.162498 36649084 PMC9977505

[B111] Nakayama A. Nakaoka H. Yamamoto K. Sakiyama M. Shaukat A. Toyoda Y. (2017). GWAS of clinically defined gout and subtypes identifies multiple susceptibility loci that include urate transporter genes. Ann. Rheumatic Dis. 76, 869–877. 10.1136/annrheumdis-2016-209632 PMC5530361 27899376

[B112] Newman D. J. Cragg G. M. (2020). Natural products as sources of new drugs over the nearly four decades from 01/1981 to 09/2019. J. Nat. Prod. 83, 770–803. 10.1021/acs.jnatprod.9b01285 32162523

[B113] Ngandeu-Singwe M. (2003). Worldwide Trends in Hyperuricaemia from 2000 to 2023: A Systematic Review and Modelling Analysis. 10.1016/S2665-9913(25)00344-3 41662849

[B114] Nguyen D. Ping F. Mu W. Hill P. Atkins R. C. Chadban S. J. (2006). Macrophage accumulation in human progressive diabetic nephropathy. Nephrol. Carlt. 11, 226–231. 10.1111/j.1440-1797.2006.00576.x 16756636

[B115] Nguyen T. B. Louie S. M. Daniele J. R. Tran Q. Dillin A. Zoncu R. (2017). DGAT1-dependent lipid droplet biogenesis protects mitochondrial function during starvation-induced autophagy. Dev. Cell 42, 9–21.e5. 10.1016/j.devcel.2017.06.003 28697336 PMC5553613

[B116] Niu H. Ren X. Tan E. Wan X. Wang Y. Shi H. (2023). CD36 deletion ameliorates diabetic kidney disease by restoring fatty acid oxidation and improving mitochondrial function. Ren. Fail. 45, 2292753. 10.1080/0886022X.2023.2292753 38097943 PMC10732185

[B117] Nowotny K. Jung T. Höhn A. Weber D. Grune T. (2015). Advanced glycation end products and oxidative stress in type 2 diabetes mellitus. Biomolecules 5, 194–222. 10.3390/biom5010194 25786107 PMC4384119

[B118] Ogiku M. Kono H. Hara M. Tsuchiya M. Fujii H. (2011). Glycyrrhizin prevents liver injury by inhibition of high-mobility group box 1 production by kupffer cells after ischemia-reperfusion in rats. J. Pharmacol. Exp. Ther. 339, 93–98. 10.1124/jpet.111.182592 21737537

[B119] Ogura Y. Kitada M. Xu J. Monno I. Koya D. (2020). CD38 inhibition by apigenin ameliorates mitochondrial oxidative stress through restoration of the intracellular NAD+/NADH ratio and Sirt3 activity in renal tubular cells in diabetic rats. Aging (Albany NY) 12, 11325–11336. 10.18632/aging.103410 32507768 PMC7343471

[B120] Ozbek E. (2012). Induction of oxidative stress in kidney. Int. J. Nephrol. 2012, 465897. 10.1155/2012/465897 22577546 PMC3345218

[B121] Pagliarini D. J. Calvo S. E. Chang B. Sheth S. A. Vafai S. B. Ong S.-E. (2008). A mitochondrial protein compendium elucidates complex I disease biology. Cell 134, 112–123. 10.1016/j.cell.2008.06.016 18614015 PMC2778844

[B122] Pal R. Bhadada S. K. (2023). AGEs accumulation with vascular complications, glycemic control and metabolic syndrome: a narrative review. Bone 176, 116884. 10.1016/j.bone.2023.116884 37598920

[B123] Pan J. Zhang C. Shi M. Guo F. Liu J. Li L. (2021). Ethanol extract of liriodendron chinense (hemsl.) sarg barks attenuates hyperuricemic nephropathy by inhibiting renal fibrosis and inflammation in mice. J. Ethnopharmacol. 264, 113278. 10.1016/j.jep.2020.113278 32841699

[B124] Panche A. N. Diwan A. D. Chandra S. R. (2016). Flavonoids: an overview. J. Nutr. Sci. 5, e47. 10.1017/jns.2016.41 28620474 PMC5465813

[B125] Patera F. Gatticchi L. Cellini B. Chiasserini D. Reboldi G. (2024). Kidney fibrosis and oxidative stress: from molecular pathways to new pharmacological opportunities. Biomolecules 14, 137. 10.3390/biom14010137 38275766 PMC10813764

[B126] Patial V. Katoch S. Chhimwal J. Dadhich G. Sharma V. Rana A. (2023). Catechins prevent obesity-induced kidney damage by modulating PPARγ/CD36 pathway and gut-kidney axis in rats. Life Sci. 316, 121437. 10.1016/j.lfs.2023.121437 36702203

[B127] Peng B. Dai J. Ji S. Yang Y. Zuo A. Xu S. (2025). Quercetin ameliorates hyperuricemic nephropathy through improving gut dysfunctions and decreasing gut bacteria-derived uremic toxins. Phytomedicine 143, 156801. 10.1016/j.phymed.2025.156801 40403599

[B128] Peti-Peterdi J. Toma I. Sipos A. Vargas S. L. (2009). Multiphoton imaging of renal regulatory mechanisms. Physiol. (Bethesda) 24, 88–96. 10.1152/physiol.00001.2009 19364911 PMC4580236

[B129] Popova M. Bankova V. (2023). Contemporary methods for the extraction and isolation of natural products. BMC Chem. 17, 68. 10.1186/s13065-023-00960-z 37391736 PMC10314546

[B130] Qi M. Wang X. Xu H. Yang Z. Cheng Y. Zhou B. (2020). Protective effect of ferulic acid on STZ-induced diabetic nephropathy in rats. Food Funct. 11, 3706–3718. 10.1039/c9fo02398d 32307498

[B131] Qiu M. Ding L.-L. Zhang M. Zhou H.-R. (2021). Safety of four SGLT2 inhibitors in three chronic diseases: a meta-analysis of large randomized trials of SGLT2 inhibitors. Diabetes Vasc. Dis. Res. 18, 14791641211011016. 10.1177/14791641211011016 PMC8481734 33887983

[B132] Qu L. Jiao B. (2023). The interplay between immune and metabolic pathways in kidney disease. Cells 12, 1584. 10.3390/cells12121584 37371054 PMC10296595

[B133] Qu T. Zhang N. Cai J. Li C. Zhang Y. Xu X. (2025). Integrated bioinformatics and multi-omics reveal astragalus extract’s gut-kidney axis mechanism in hyperuricemic nephropathy *via* purine metabolism. Phytomedicine 146, 157121. 10.1016/j.phymed.2025.157121 40774007

[B134] Radhakrishnan A. Goldstein J. L. McDonald J. G. Brown M. S. (2008). Switch-like control of SREBP-2 transport triggered by small changes in ER cholesterol: a delicate balance. Cell Metab. 8, 512–521. 10.1016/j.cmet.2008.10.008 19041766 PMC2652870

[B135] Ratliff B. B. Abdulmahdi W. Pawar R. Wolin M. S. (2016). Oxidant mechanisms in renal injury and disease. Antioxid. Redox Signal 25, 119–146. 10.1089/ars.2016.6665 26906267 PMC4948213

[B136] Régnier M. Van Hul M. Knauf C. Cani P. D. (2021). Gut microbiome, endocrine control of gut barrier function and metabolic diseases. J. Endocrinol. 248, R67–R82. 10.1530/JOE-20-0473 33295880

[B137] Rong J. Zhang Z. Peng X. Li P. Zhao T. Zhong Y. (2024). Mechanisms of hepatic and renal injury in lipid metabolism disorders in metabolic syndrome. Int. J. Biol. Sci. 20, 4783–4798. 10.7150/ijbs.100394 39309427 PMC11414397

[B138] Rossing P. Caramori M. L. Chan J. C. N. Heerspink H. J. L. Hurst C. Khunti K. (2022). KDIGO 2022 clinical practice guideline for diabetes management in chronic kidney disease. Kidney Int. 102, S1–S127. 10.1016/j.kint.2022.06.008 36272764

[B139] Sakashita M. Tanaka T. Inagi R. (2021). Metabolic changes and oxidative stress in diabetic kidney disease. Antioxidants (Basel) 10, 1143. 10.3390/antiox10071143 34356375 PMC8301131

[B140] Salaritabar A. Darvishi B. Hadjiakhoondi F. Manayi A. Sureda A. Nabavi S. F. (2017). Therapeutic potential of flavonoids in inflammatory bowel disease: a comprehensive review. World J. Gastroenterol. 23, 5097–5114. 10.3748/wjg.v23.i28.5097 28811706 PMC5537178

[B141] Saldívar-González F. I. Aldas-Bulos V. D. Medina-Franco J. L. Plisson F. (2022). Natural product drug discovery in the artificial intelligence era. Chem. Sci. 13, 1526–1546. 10.1039/d1sc04471k 35282622 PMC8827052

[B142] Sattarinezhad A. Roozbeh J. Shirazi Yeganeh B. Omrani G. R. Shams M. (2019). Resveratrol reduces albuminuria in diabetic nephropathy: a randomized double-blind placebo-controlled clinical trial. Diabetes & Metabolism 45, 53–59. 10.1016/j.diabet.2018.05.010 29983230

[B143] Schlesinger N. Padnick-Silver L. LaMoreaux B. (2022). Enhancing the response rate to recombinant uricases in patients with gout. BioDrugs 36, 95–103. 10.1007/s40259-022-00517-x 35316517 PMC8938732

[B144] Shahidi F. Yeo J. (2018). Bioactivities of phenolics by focusing on suppression of chronic diseases: a review. Int. J. Mol. Sci. 19, 1573. 10.3390/ijms19061573 29799460 PMC6032343

[B145] Sinha S. K. Nicholas S. B. (2023). Pathomechanisms of diabetic kidney disease. JCM 12, 7349. 10.3390/jcm12237349 38068400 PMC10707303

[B146] Su W. Cao R. Zhang X. Guan Y. (2020). Aquaporins in the Kidney: Physiology and Pathophysiology. 10.1152/ajprenal.00304.2019 31682170

[B147] Summers S. Quimby J. (2024). Insights into the gut-kidney axis and implications for chronic kidney disease management in cats and dogs. Vet. J. 306, 106181. 10.1016/j.tvjl.2024.106181 38897377

[B148] Szeto H. H. Liu S. Soong Y. Alam N. Prusky G. T. Seshan S. V. (2016). Protection of mitochondria prevents high-fat diet-induced glomerulopathy and proximal tubular injury. Kidney Int. 90, 997–1011. 10.1016/j.kint.2016.06.013 27519664

[B149] Tang S. C. W. Yiu W. H. (2020). Innate immunity in diabetic kidney disease. Nat. Rev. Nephrol. 16, 206–222. 10.1038/s41581-019-0234-4 31942046

[B150] Thakur V. Nargis S. Gonzalez M. Pradhan S. Terreros D. Chattopadhyay M. (2017). Role of glycyrrhizin in the reduction of inflammation in diabetic kidney disease. Nephron 137, 137–147. 10.1159/000477820 28641285

[B151] Thomford N. E. Senthebane D. A. Rowe A. Munro D. Seele P. Maroyi A. (2018). Natural products for drug discovery in the 21st century: innovations for novel drug discovery. Int. J. Mol. Sci. 19, 1578. 10.3390/ijms19061578 29799486 PMC6032166

[B152] Toyoki D. Shibata S. Kuribayashi-Okuma E. Xu N. Ishizawa K. Hosoyamada M. (2017). Insulin stimulates uric acid reabsorption *via* regulating urate transporter 1 and ATP-Binding cassette subfamily G member 2. Am. J. Physiology-Renal Physiology 313, F826–F834. 10.1152/ajprenal.00012.2017 28679589

[B153] Triplitt C. L. (2012). Understanding the kidneys’ role in blood glucose regulation. Am. J. Manag. Care 18, S11–S16. 22559853

[B154] Tsuji K. Uchida N. Nakanoh H. Fukushima K. Haraguchi S. Kitamura S. (2024). The gut–kidney axis in chronic kidney diseases. Diagnostics 15, 21. 10.3390/diagnostics15010021 39795549 PMC11719742

[B155] Tsushima Y. Nishizawa H. Tochino Y. Nakatsuji H. Sekimoto R. Nagao H. (2013). Uric acid secretion from adipose tissue and its increase in obesity. J. Biol. Chem. 288, 27138–27149. 10.1074/jbc.M113.485094 23913681 PMC3779712

[B156] Vanaie A. Shahidi S. Iraj B. Siadat Z. D. Kabirzade M. Shakiba F. (2019). Curcumin as a major active component of turmeric attenuates proteinuria in patients with overt diabetic nephropathy. J. Res. Med. Sci. 24, 77. 10.4103/jrms.JRMS_1055_18 31523263 PMC6734668

[B157] Wada J. Makino H. (2013). Inflammation and the pathogenesis of diabetic nephropathy. Clin. Sci. (Lond) 124, 139–152. 10.1042/CS20120198 23075333

[B158] Wang X. C. Liu C. H. Chen Y. J. Wu Y. Yang L. S. Liu H. M. (2013). Clinical and pathological analysis of the kidney in patients with hypertensive nephropathy. Exp. Ther. Med. 6, 1243–1246. 10.3892/etm.2013.1297 24223652 PMC3820837

[B159] Wang J. Liu H. Li N. Zhang Q. Zhang H. (2014). The protective effect of fucoidan in rats with streptozotocin-induced diabetic nephropathy. Mar. Drugs 12, 3292–3306. 10.3390/md12063292 24886867 PMC4071577

[B160] Wang Y. Hu B. Feng S. Wang J. Zhang F. (2020). Target recognition and network pharmacology for revealing anti-diabetes mechanisms of natural product. J. Comput. Sci. 45, 101186. 10.1016/j.jocs.2020.101186

[B161] Wang D. Yin L. Chen R. Tan W. Liang L. Xiang J. (2022a). Senescent renal tubular epithelial cells activate fibroblasts by secreting shh to promote the progression of diabetic kidney disease. Front. Med. (Lausanne) 9, 1018298. 10.3389/fmed.2022.1018298 36760880 PMC9905119

[B162] Wang M. Lu S. Zhao H. Liu Z. Sheng K. Fang J. (2022b). Natural polysaccharides as potential anti-fibrotic agents: a review of their progress. Life Sci. 308, 120953. 10.1016/j.lfs.2022.120953 36103957

[B163] Wang M. Pang Y. Guo Y. Tian L. Liu Y. Shen C. (2022c). Metabolic reprogramming: a novel therapeutic target in diabetic kidney disease. Front. Pharmacol. 13, 970601. 10.3389/fphar.2022.970601 36120335 PMC9479190

[B164] Wang H. Ainiwaer A. Song Y. Qin L. Peng A. Bao H. (2023a). Perturbed gut microbiome and fecal and serum metabolomes are associated with chronic kidney disease severity. Microbiome 11, 3. 10.1186/s40168-022-01443-4 36624472 PMC9827681

[B165] Wang Y. Jin M. Cheng C. K. Li Q. (2023b). Tubular injury in diabetic kidney disease: molecular mechanisms and potential therapeutic perspectives. Front. Endocrinol. 14, 1238927. 10.3389/fendo.2023.1238927 PMC10433744 37600689

[B166] Wang Z. Wu G. Niu T. Guo Y. Wang C. Wang X. (2024). Polysaccharide isolated from dioscorea septemloba improves hyperuricemia and alleviates renal fibrosis through gut-kidney axis in mice. Int. J. Biol. Macromol. 282, 137112. 10.1016/j.ijbiomac.2024.137112 39489240

[B167] Wang Y.-A. Guo X. Zhang M.-Q. Sun S.-T. Ren Q.-D. Wang M.-X. (2025). Evaluation of anti-hyperuricemic and nephroprotective activities and discovery of new XOD inhibitors of morus alba L. root bark. J. Ethnopharmacol. 343, 119476. 10.1016/j.jep.2025.119476 39938762

[B168] Wang Y. Huang B. Wei X. Guan Y. Li L. Zheng Y. (2026). Gut microbiota metabolic reprogramming drives the development of metabolic diseases in the host. Gut Microbes 18, 2644681. 10.1080/19490976.2026.2644681 41830551 PMC12990950

[B169] Wen S. Arakawa H. Tamai I. (2024). Uric acid in health and disease: from physiological functions to pathogenic mechanisms. Pharmacol. & Ther. 256, 108615. 10.1016/j.pharmthera.2024.108615 38382882

[B170] Wen X. Zhang X. Wang W. Zhao F. Xie M. Pei G. (2025). Effects of holothuria leucospilota polysaccharide on alleviating diabetic kidney disease through regulating inflammation. Int. J. Biol. Macromol. 306, 142027. 10.1016/j.ijbiomac.2025.142027 40081694

[B171] Wong J. Piceno Y. M. DeSantis T. Z. Pahl M. Andersen G. L. Vaziri N. D. (2014). Expansion of urease- and uricase-containing, indole- and p-cresol-forming and contraction of short-chain fatty acid-producing intestinal microbiota in ESRD. Am. J. Nephrol. 39, 230–237. 10.1159/000360010 24643131 PMC4049264

[B172] Wright E. M. (2013). Glucose transport families SLC5 and SLC50. Mol. Asp. Med. 34, 183–196. 10.1016/j.mam.2012.11.002 23506865

[B173] Wu X.-Q. Zhang D.-D. Wang Y.-N. Tan Y.-Q. Yu X.-Y. Zhao Y.-Y. (2021). AGE/RAGE in diabetic kidney disease and ageing kidney. Free Radic. Biol. Med. 171, 260–271. 10.1016/j.freeradbiomed.2021.05.025 34019934

[B174] Wu Y. Liang Y. Liang J. Sun W. Zhang Q. Li M. (2026). Systematic deciphering of ATBC nephrotoxicity mechanisms *via* machine learning and single‐cell analysis. Med. Bull. 2, 141–154. 10.1002/mdb2.70027

[B175] Xu K.-Y. Xia G.-H. Lu J.-Q. Chen M.-X. Zhen X. Wang S. (2017). Impaired renal function and dysbiosis of gut microbiota contribute to increased trimethylamine-N-oxide in chronic kidney disease patients. Sci. Rep. 7, 1445. 10.1038/s41598-017-01387-y 28469156 PMC5431124

[B176] Xu X. Zhu R. Ying J. Zhao M. Wu X. Cao G. (2020). Nephrotoxicity of herbal medicine and its prevention. Front. Pharmacol. 11, 569551. 10.3389/fphar.2020.569551 33178019 PMC7593559

[B177] Xu J. Weng Z. Cui Q. Yu W. Lin Y. Song H. (2025). Current research on the regulation of glycolipid metabolism by plant-derived active polysaccharides. Trends Food Sci. & Technol. 159, 104959. 10.1016/j.tifs.2025.104959

[B178] Yahya N. A. Attan N. Wahab R. A. (2018). An overview of cosmeceutically relevant plant extracts and strategies for extraction of plant-based bioactive compounds. Food Bioprod. Process. 112, 69–85. 10.1016/j.fbp.2018.09.002

[B179] Yamagishi S. (2011). Role of advanced glycation end products (AGEs) and receptor for AGEs (RAGE) in vascular damage in diabetes. Exp. Gerontol. 46, 217–224. 10.1016/j.exger.2010.11.007 21111800

[B180] Yan S.-F. Ramasamy R. Bucciarelli L. G. Wendt T. Lee L. K. Hudson B. I. (2004). RAGE and its ligands: a lasting memory in diabetic complications? Diab. Vasc. Dis. Res. 1, 10–20. 10.3132/dvdr.2004.001 16305050

[B181] Yan Z.-J. Han X.-Y. Zhang L. Wang Q. Kang Y. Yuan Y. (2025). Advances in traditional Chinese medicine for endocrine-metabolic diseases in 2024. Tradit. Med. Res. 10, 69. 10.53388/tmr20250211001

[B182] Yang J. Dong H. Wang Y. Jiang Y. Zhang W. Lu Y. (2020). Cordyceps cicadae polysaccharides ameliorated renal interstitial fibrosis in diabetic nephropathy rats by repressing inflammation and modulating gut microbiota dysbiosis. Int. J. Biol. Macromol. 163, 442–456. 10.1016/j.ijbiomac.2020.06.153 32592781

[B183] Yang X. Jiang D. Wang Y. Duan M. Huang Y. Wang X. (2024). Chlorogenic acid alleviates renal fibrosis by reducing lipid accumulation in diabetic kidney disease through suppressing the Notch1 and Stat3 signaling pathway. Ren. Fail. 46, 2371988. 10.1080/0886022x.2024.2371988 38952291 PMC11221469

[B184] Yi H. Jiang Y. Li W. Shen L. Zhang W. Li S. (2025). Scutellarin prevents obesity-induced renal fibrosis *via* reduced activation of AP-1. J. Transl. Med. 23, 611. 10.1186/s12967-025-06616-x 40457446 PMC12131579

[B185] Yoon H. Shaw J. L. Haigis M. C. Greka A. (2021). Lipid metabolism in sickness and in health: emerging regulators of lipotoxicity. Mol. Cell 81, 3708–3730. 10.1016/j.molcel.2021.08.027 34547235 PMC8620413

[B186] Zeisberg M. Kalluri R. (2015). Physiology of the renal interstitium. Clin. J. Am. Soc. Nephrol. 10, 1831–1840. 10.2215/CJN.00640114 25813241 PMC4594057

[B187] Zeng M. Yang L. He D. Li Y. Shi M. Zhang J. (2017). Metabolic pathways and pharmacokinetics of natural medicines with low permeability. Drug Metab. Rev. 49, 464–476. 10.1080/03602532.2017.1377222 28911247

[B188] Zhai S.-Y. Li L.-R. Jia J.-H. Cao Y.-X. Li M.-Y. Zheng Y.-F. (2025). Food and medicine homology components ameliorate metabolic diseases *via* dual pathways: blood absorption and nonabsorptive pathways. 2, 9420133.

[B189] Zhang Y.-J. Li S. Gan R.-Y. Zhou T. Xu D.-P. Li H.-B. (2015). Impacts of gut bacteria on human health and diseases. Int. J. Mol. Sci. 16, 7493–7519. 10.3390/ijms16047493 25849657 PMC4425030

[B190] Zhang Q.-W. Lin L.-G. Ye W.-C. (2018). Techniques for extraction and isolation of natural products: a comprehensive review. Chin. Med. 13, 20. 10.1186/s13020-018-0177-x 29692864 PMC5905184

[B191] Zhang B. Zhang X. Zhang C. Shen Q. Sun G. Sun X. (2019). Notoginsenoside R1 protects db/db mice against diabetic nephropathy *via* upregulation of Nrf2-mediated HO-1 expression. Molecules 24, 247. 10.3390/molecules24020247 30634720 PMC6359411

[B192] Zhang X. Agborbesong E. Li X. (2021). The role of mitochondria in acute kidney injury and chronic kidney disease and its therapeutic potential. Int. J. Mol. Sci. 22, 11253. 10.3390/ijms222011253 34681922 PMC8537003

[B193] Zhang M. Yang L. Zhu M. Yang B. Yang Y. Jia X. (2022). Moutan cortex polysaccharide ameliorates diabetic kidney disease *via* modulating gut microbiota dynamically in rats. Int. J. Biol. Macromol. 206, 849–860. 10.1016/j.ijbiomac.2022.03.077 35307460

[B194] Zhang J.-L. Du C. Poon C. C.-W. He M.-C. Wong M.-S. Wang N.-N. (2023a). Structural characterization and protective effect against renal fibrosis of polysaccharide from ligustrum lucidum ait. J. Ethnopharmacol. 302, 115898. 10.1016/j.jep.2022.115898 36372193

[B195] Zhang Y. Li Y. Li C. Zhao Y. Xu L. Ma S. (2023b). Paeonia × suffruticosa Andrews leaf extract and its main component apigenin 7-O-glucoside ameliorate hyperuricemia by inhibiting xanthine oxidase activity and regulating renal urate transporters. Phytomedicine 118, 154957. 10.1016/j.phymed.2023.154957 37478683

[B196] Zhang A. Wang J. Hu Y. Qiu Y. Dong C. (2024a). Polysaccharides play an anti-fibrotic role by regulating intestinal flora: a review of research progress. Int. J. Biol. Macromol. 271, 131982. 10.1016/j.ijbiomac.2024.131982 38724335

[B197] Zhang X. Wang J. Xiang S. Zhao L. Lv M. Duan Y. (2024b). Astragaloside I from *astragalus* attenuates diabetic kidney disease by regulating HDAC3/klotho/TGF-**β**1 loop. Am. J. Chin. Med. 52, 1795–1817. 10.1142/s0192415x24500708 39347955

[B198] Zhang Y. Deng Y. Yang Y. Yang Z. Yin Y. Xie J. (2024c). Polysaccharides from dendrobium officinale delay diabetic kidney disease interstitial fibrosis through LncRNA XIST/TGF-β1. Biomed. & Pharmacother. 175, 116636. 10.1016/j.biopha.2024.116636 38677245

[B199] Zhang L. Jiang L. Xu R. Zhang X. Zhang B. Yue R. (2025a). Epidemiological research on diabetic nephropathy at global, regional, and national levels from 1990 to 2021: an analysis derived from the global burden of disease 2021 study. Front. Endocrinol. 16, 1647064. 10.3389/fendo.2025.1647064 PMC12420290 40937407

[B200] Zhang Y. He F. Yu X. Li T. Zhou L. Shen B. (2025b). Hyperuricemia-induced kidney injury: a narrative review of mechanisms and therapeutic advances. BMC Nephrol. 26, 629. 10.1186/s12882-025-04414-7 41225371 PMC12613580

[B201] Zhang Y. Hao R. Zhong Q. Han M. Zhao S. Sun X. (2026). Mechanistic insights into the regulation of glucose‒lipid metabolism by the bioactive constituents of ginseng. J. Ginseng Res. 50, 100928. 10.1016/j.jgr.2025.12.001 41788585 PMC12959281

[B202] Zhao H. Yang C.-E. Liu T. Zhang M.-X. Niu Y. Wang M. (2023a). The roles of gut microbiota and its metabolites in diabetic nephropathy. Front. Microbiol. 14, 1207132. 10.3389/fmicb.2023.1207132 37577423 PMC10413983

[B203] Zhao T. Yang M. Ma L. Liu X. Ding Q. Chai G. (2023b). Structural modification and biological activity of polysaccharides. Molecules 28, 5416. 10.3390/molecules28145416 37513287 PMC10384959

[B204] Zhao D.-M. Zhong R. Wang X.-T. Yan Z.-H. (2024). Mitochondrial dysfunction in diabetic nephropathy: insights and therapeutic avenues from traditional Chinese medicine. Front. Endocrinol. (Lausanne) 15, 1429420. 10.3389/fendo.2024.1429420 39109083 PMC11300275

[B205] Zhou X. Zhang B. Zhao X. Lin Y. Zhuang Y. Guo J. (2022). Chlorogenic acid prevents hyperuricemia nephropathy *via* regulating TMAO-related gut microbes and inhibiting the PI3K/AKT/mTOR pathway. J. Agric. Food Chem. 70, 10182–10193. 10.1021/acs.jafc.2c03099 35950815

[B206] Zhu Y. Ouyang Z. Du H. Wang M. Wang J. Sun H. (2022). New opportunities and challenges of natural products research: when target identification meets single-cell multiomics. Acta Pharm. Sin. B 12, 4011–4039. 10.1016/j.apsb.2022.08.022 36386472 PMC9643300

[B207] Zhu L.-R. Li S.-S. Zheng W.-Q. Ni W.-J. Cai M. Liu H.-P. (2023). Targeted modulation of gut microbiota by traditional Chinese medicine and natural products for liver disease therapy. Front. Immunol. 14, 1086078. 10.3389/fimmu.2023.1086078 36817459 PMC9933143

