# Correction: Multidimensional mechanisms of natural products in modulating metabolic dysfunction–associated kidney disease: recent advances and future perspectives

**DOI:** 10.3389/fphar.2026.1927694

**Published:** 2026-07-22

**Authors:** Yue Zhang, Jiarui Li, Qi Zhong, Mingda Han, Xiao Sun, Huili Huang, Shuang Zhao, Hao Xu, Kaile Ma, Xiangyan Li, Min Li

**Affiliations:** 1 Guang’anmen Hospital, China Academy of Chinese Medical Sciences, Beijing, China; 2 College of Traditional Chinese Medicine, Changchun University of Chinese Medicine, Changchun, China; 3 Research Laboratory of Molecular Biology, Guang’anmen Hospital, China Academy of Chinese Medical Sciences, Beijing, China

**Keywords:** diabetic kidney disease, hyperuricemic nephropathy, metabolic dysfunction–associated kidney disease, natural products, obesity-related glomerulopathy, targets

In the Funding statement, a funder was erroneously omitted. The research was supported by the Clinical Research Center for Metabolic Diseases (Integrated Traditional Chinese and Western Medicine) of Chongqing (Grant No. SQMS2026DXB-002). In addition, an incorrect project number was provided for the project for the development of the “Guidelines for the Diagnosis and Treatment of Type 2 Diabetes with Integrated Traditional Chinese and Western Medicine” from the National Administration of Traditional Chinese Medicine. The correct project number is ZYZB-2022-798.

The Funding statement has been corrected and updated as follows: The author(s) declared that financial support was received for this work and/or its publication. This work was supported by the Clinical Research Center for Metabolic Diseases (Integrated Traditional Chinese and Western Medicine) of Chongqing (Grant No. SQMS2026DXB-002) and the Project for the Development of the “Guidelines for the Diagnosis and Treatment of Type 2 Diabetes with Integrated Traditional Chinese and Western Medicine” from the National Administration of Traditional Chinese Medicine (Project No. ZYZB-2022-798).

The original version of this article has been updated.

